# Effectiveness of a pedagogical module for the process of weaning from mechanical ventilation in advanced nursing education

**DOI:** 10.1371/journal.pone.0332792

**Published:** 2026-06-29

**Authors:** Sakinah Awangharun, Norlidah Alias

**Affiliations:** 1 Medical Education Research and Development Unit, Faculty of Medicine University of Malaya, Kuala Lumpur, Malaysia; 2 Department of Curriculum and Instructional Technology, Faculty of Education, University of Malaya, Kuala Lumpur, Malaysia; University of Huelva: Universidad de Huelva, SPAIN

## Abstract

**Background:**

Pedagogical module for weaning process from mechanical ventilation (WPMV) is the specific need for advanced nursing education in cardiothoracic intensive care unit (CICU). The CICU nurses need to have Advanced nursing personnel in the CICU require comprehensive theoretical knowledge and applied clinical reasoning skills including patient data analysis, structural decision-making, and care decision justifications to safely adjust ventilator settings following cardiothoracic surgery. However, currently no specific a pedagogical module for advanced nursing education used for CICU in Malaysia.

**Aim:**

To investigate the effectiveness of a structured pedagogical module for WPMV in improving knowledge and applied clinical reasoning among Cardiothoracic Intensive Care Unit (CICU) nurses.

**Methods:**

This study utilizes a quasi-experimental, non-randomized pre-post design. A total of 24 CICU nurses participated. Participants were grouped based on an institutional competency threshold of 70%, which reflects the standard passing criterion for clinical assessments in the study setting. The 70% cutoff represents the standard institutional passing threshold established by the Medical Education and Nursing Division at the National Heart Institute (IJN) for postgraduate clinical assessments and unit privilege. This study focuses primarily on within-group changes following the intervention, while the comparison group is used as a performance benchmark rather than for inferential comparison.

**Results:**

The implementation of the pedagogical module led to a statistically significant improvement in participants’ knowledge and applied clinical reasoning. Participants in the intervention group demonstrated a statistically significant improvement in theoretical scores (mean difference = 37.63, p < 0.001). The large effect size indicates substantial educational impact. Due to the small and unequal group sizes, between-group comparisons were interpreted descriptively rather than inferentially.

**Conclusion:**

The WPMV pedagogical module significantly improves theoretical knowledge and applied clinical reasoning among CICU nurses. However, conclusions are limited to educational outcomes, and further research is required to evaluate its impact on clinical practice and patient outcomes.

## Introduction

For the first five decades of the 20th century, the job scope and function of nurses become increasingly sophisticated currently. The roles and responsibilities of nurses working in the CICU are rapidly changing and this creates an urgent need for the education health professionals and nurses in particular to specialization [1].

Currently, in Malaysia WPMV in practice uses the protocol and clinical practice guideline in assisting nurses and doctors in performing the procedure. Traditionally, CICU Nurses perform the WPMV procedure through evidence-based practice. The protocol and clinical practice guideline were used as on job training guide as well for CICU nurses and doctor. Then, the organization were credentialing and privileging all the CICU nurses after on job training to prevent them from any blurry boundaries in practice (illegality), past and present [2,3].

In ensuring quality and standard nursing education system in Malaysia, the development of a pedagogical module for the process of weaning from mechanical ventilation in advanced nursing education is crucial. This framework serves as a proactive educational strategy designed to increase knowledge and clinical reasoning skills, thereby minimizing the underlying factors associated with future legal, medico-legal, and malpractice risks among nurses working in cardiothoracic intensive care units (CICU) [4–7].

The weaning process from MV is extremely risky if the nurses on duty do not have advanced knowledge and skills in managing patients on MV. The CICU nurses need to have high knowledge of the weaning process from MV for machine care knowledge with patient management as well as the decision to change the mode and setting of ventilation mechanism with patient use after Cardiothoracic surgery [8].

The majority post operation cardiac surgery patients require support from mechanical ventilation to their respiratory system. This respiratory support is fully required for all unconscious patient post operation cardiac surgery; it is due to an anaesthetic effect given before the patient undergoes surgery [9]. Practically, this specialized nursing education in intensive care requires knowledge in keeping with doctors as it were more helpful for in-patient management and treatment procedures as well as nursing care [10]. So, if a nurse has not undergone advanced nursing education (subspecialties education), these practices have been going on without any guideline, or any evidence practice supporting the nurses’ role and responsibilities in actively doing this procedure. Because of shortage of doctors (cardiac Anaesthesiologist/ Intensivist), the CICU nurses have assisted in the weaning process to ensure the medical decision on the patient situation can be expedited effectively [11].

The CICU nurses perform and learn WPMV procedure through unstructured traditional education training. This unstructured teaching and learning is known as work-based education; this refers to all forms of learning occurring in the real practice environment. It has traditionally been the practice in the CICU environment and other healthcare education. Work-based learning provides individuals with the skills needed to successfully find and keep jobs, and to advance their career. WPMV is one example of education and training needs in the CICU and traditionally has been practised as work-based learning. However, this is a big challenge to them if they are facing abnormal flow as per protocol in practice that required them to consult with doctors all the time and may cause delay in the WPMV process. Delaying the process of WPMV may possibly cause complications. Meanwhile, the CICU nurses learn through individual experience on the job in critical thinking, decision making and decision judgment while performing in managing ventilated patient of post cardiothoracic surgery in order to ensure number of factors associated with successful weaning. The questions they consider are: (1) has the underlying condition improved? (2) is the patient’s general condition optimal? (3) have potential airway problems been identified and remedied? and lastly (4) is breathing adequate?. Therefore, in answering these questions, in current practice the CICU staff nurse with minimal knowledge and skills staff nurses should have extra sense in predicting which patients are able to be weaned based on individual experience until the patient is able to extubate or unplanned extubation [12]. Practically, there is no pedagogical module in WPMV in CICU at Institut Jantung Negara (IJN) currently.

However, teaching facilitating learning has been conducted by unstructured training through reflective practice and mentor mentee guided for novice in CICU for quality standard and patient safety. Basically, the content on the WPMV subject is really important to emphasize as daily routine when nurses receive post cardiac surgery patients every day. Besides that, another issue are lack of pedagogical knowledge and skills among instructor since they are practitioner in CICU. The instructors may have minimal knowledge in pedagogical principles since they are not trained as formal teachers in playing full roles and responsibilities.

The theoretical framework of this research integrates three core models to guide the module development process (S1 Fig). This framework was constructed by evaluating curriculum design from both theoretical and practical perspectives by referring to the model related to curriculum from view of theory and practice. This study has selected three models consisting of Johns Model of Reflection (1995), Taba Model (1962), and Gagne’s Model (1958). The discussion of theoretical framework begins with the Model of Reflection by Johns (1995) using Carper’s Ways of Knowing to assist CICU nurses in the process implementation stage and used during training. As a part of guidance in developing a WPMV module for nursing education used the Taba Model. Gagne’s Model was used during the planning process of developing the module; it proposed a series of events that follow a systematic instructional design process based on the behaviourist approach to learning, with a focus on the outcomes or behaviours of instruction or training.

The reflective model assists the learning process in nursing education because it required nurses to recall what they are doing in practice pre, during and post while working in the CICU. The models help enhance nursing education gradually and nurses always can gain a lot of knowledge through reflective learning. Therefore, this practice is very helpful when incorporated in the WPMV module. By reflecting on practice, nurses were share the ideas that require for insertion in the module weaning process from MV. To sustain the standard practice in the weaning process at the CICU, collaboration with CICU colleagues in the hospital was appreciated in order to assure safe practice [13,14].

The models function as guidance for the researcher in developing new modules. This study selected the Taba model for guidance in developing the WPMV module. Taba took a grassroots approach to curriculum development. In this study, the Taba model combined five common elements such as objectives, content, learning activities, WPMV strategies and evaluative measures [15].

Utilising an expert with knowledge and experience on this subject is beneficial to the new learning effective in the health care setting. It is stronger to have evidence when the standard of practice is supported by a strong theoretical model. This study will use the panel of expert’s opinion as a consensus in developing and designing the curriculum of a WPMV module for ANE. In designing and developing the WPMV module, eight steps were required in the Taba model. Taba favoured an inductive approach to curriculum development, beginning with specifics and ending with a general view [16].

An eight-step sequence operates in the process of developing and designing curriculum following the Taba guide. First, producing pilot unit representative of the grade level or subject area.

The following are the eight-steps sequence for curriculum development for producing the pilot unit used as a guide when developing the curriculum. The eight-steps sequence start with diagnose needs, formulate specific objectives, select of content, organizing of content, selection of learning experiences, organization of learning activities, determination of what to evaluate whether objectives have been achieved and lastly checking for balancing and sequence (S2 Fig) [17].

Furthermore, Gagne’s model of instructional design is based on the information processing model of the mental events occurring when adults process various stimuli; it focuses on the learning outcomes and how to arrange specific instructional events to achieve those outcomes [18]. Applying Gagne’s nine-step model ensures effective and systematic learning since it structures the lesson plans and provides a holistic view of the WPMV. This study has chosen the weaning process and were use Gagne’s “events of instruction” to design a lesson plan for this subject as shown in (S1 Table).

### Research objective

To evaluate the effectiveness of a structured pedagogical module for WPMV in improving the knowledge and applied clinical reasoning of Cardiothoracic Intensive Care Unit (CICU) nurses.

### Research question

How effective is a structured pedagogical module for WPMV in improving the knowledge and applied clinical reasoning of CICU nurses?

## Materials and methods

### Ethical consideration

Ethical approval was obtained on 27th June 2019 with approval number IJN, ID IJNREC/417/219.

### Method

This research followed the design and development Research (DDR) Framework (Richey and Klein, 2007). This study employed a quasi-experimental pre–post design without randomisation to evaluate the effectiveness of the pedagogical module (PM) for the weaning process from mechanical ventilation (WPMV) (S3 Fig). Due to operational constraints within the clinical setting, participants could not be randomly assigned. Therefore, the study focuses on within-group changes following the intervention, while the comparison group is used as a performance benchmark rather than for inferential comparison. The PM for WPMV was implemented with 2 instructor and 24 CICU nurses over 6 weeks (S4 Fig).

### Participants and sampling

A purposive sample of 24 CICU nurses from the National Heart Institute (IJN), Malaysia, was recruited. Eligible participants held a bachelor’s degree in nursing with three years or less of specialized critical care experience. Cohort assignment was guided strictly by an absolute, criterion-referenced evaluation based on an institutional 70% passing threshold. This threshold directly mirrors non-negotiable BLS and ACLS cognitive safety cut-offs mandated for independent clinical practice.

Consequently, learners scoring under 70% formed the intervention cohort (*n* = 19), while proficient peers scoring 70% or above formed a small comparison cohort (*n* = 5). This latter cohort consisted of skilled CICU staff nurses with identical academic backgrounds (bachelor’s degree) and experience levels, serving exclusively as a descriptive baseline performance benchmark rather than for inferential comparison. Participant grouping in S2 Table was guided strictly by an absolute, criterion-referenced evaluation model rather than an arbitrary threshold

### Instructional framework and module development

The WPMV module was developed by integrating three theoretical model to ensure a systematic educational approach (S1 Fig).

1. Taba Model: Used for curriculum design, following an eight-step inductive sequence: diagnosing needs, formulating objectives, selecting and organizing content, selecting and organizing learning experiences and determining evaluation measures.
2. Gagne’s Nine Event of Instruction; Applied to structure the lesson plans and arrange instructional event to achieved specific learning outcomes.
3. Johns’ Model of reflection: Incorporated to assist nurses in recalling and evaluating their clinical practice, pre-, during and post procedure

### Instruments

The primary instruments consisted of structured theoretical assessments were designed to measure:

- Knowledge of WPMV processes
- Advanced of weaning mechanisms
- Higher-order cognitive skills, including critical thinking, decision-making and clinical judgement and applied clinical reasoning skills during the WPMV procedure (including analysis of patient data, clinical decision-making, and justification of care decisions).

In additional to quantitative test, a semi-structured interview was conducted face-to-face with two instructor post-implementation to gather qualitative on the module usability.

### Data collection

The implementation phase of the study spanned six weeks. During the first week, a pre-test was administered to all 24 participants to establish baseline scores and assign individuals to either the treatment or comparison group based on the pre-determined cut-off score. From weeks two through five, the treatment group underwent the WPMV pedagogical intervention, which was structured according to Gagné’s Nine Events of Instruction. Finally, in week six, a post-test was conducted for both the treatment and comparison groups to evaluate achievement improvement and investigate the effectiveness of the module [19].

Quantitative data were analyzed using IBM SPSS version 27. Descriptive statistics, including mean scores and standard deviations, were calculated to summarize theoretical pre-test and post-test trends. To evaluate within-group improvements, normality testing was systematically performed using the Kolmogorov-Smirnov and Shapiro-Wilk tests. Because the Shapiro-Wilk test indicated a non-normal distribution for the baseline pre-test scores (*p* = 0.015), the non-parametric Related-Samples Wilcoxon Signed-Rank Test was prioritized as the primary inferential tool to evaluate changes in medians. A parametric paired-sample *t*-test was conducted as a secondary, complementary analysis to demonstrate mean distribution transformations alongside a calculated Cohen’s *d* effect size to capture practical educational impact [19].

Pretest was conducted in week 1 before implementation pedagogical module WPMV programme. CICU nurses have sat for the pre-test in the first stage and following process the researcher needed to rank them based on data analysis skill. The researcher decided for data analysis to use a cut-off point score of 70 to determine weak and skilled students. Results cut off point score for students who have less than 70 score in pre-test are placed as weak students in the group, while those scoring above 70 points are placed in the skilled or comparison group [19]. Therefore, total number of samples in treatment is 19 students in the treatment group and 5 students in the comparison group for the study implementation phase [19] as shown in (S2 and S3 Tables).

In S2 Table. criteria Participant grouping was guided strictly by an absolute, criterion-referenced evaluation model rather than an arbitrary threshold. The 70% cutoff represents the standard institutional passing threshold established by the Medical Education and Nursing Division at the National Heart Institute (IJN) for postgraduate clinical assessments and unit privileging. This cut-score directly mirrors standard international healthcare certification models such as the mandatory cognitive performance thresholds required in Basic Life Support (BLS) and Advanced Cardiovascular Life Support (ACLS) training where an absolute baseline score (typically between 70% and 84%) is non-negotiable mandated to establish minimum clinical safety before independent practice. Learners obtaining a score below 70% were identified as requiring targeted development and placed in the intervention cohort (*n* = 19), while proficient peers (*n* = 5) served exclusively as a baseline descriptive performance benchmark S3 Table.

Both the treatment group and comparison group had sat the pretest. Post-test was carried out on both groups after the treatment group had completed the intervention.

Post test was conducted at the end of week 6 after the intervention to investigate the effectiveness based on pre-test and post-test results.

## Results

The implementation of the pedagogical module led to a statistically significant improvement in participants’ theoretical knowledge and applied clinical reasoning. Because baseline pre-test scores exhibited non-normality (*p* = 0.015), within-group improvement was evaluated primarily via the Related-Samples Wilcoxon Signed-Rank Test, which strongly confirmed a significant positive median shift between the pre-test and post-test phases (*p* = 0.001), leading to a decisive rejection of the null hypothesis. Complementary parametric analysis showed a corresponding shift in mean scores from 52.89% (±SD 10.18) to 90.53% (±SD 7.97), yielding a mean difference of 37.63 (*t* (8) = 14.30, p < 0.001). The calculated Cohen’s *d* point estimate of 3.28 indicated a very large educational effect size, confirming substantial practical significance for advanced nursing readiness. The case processing summary for the theoretical pre-test and post-test is presented in S4 Table.

The total of candidate in the treatment group (*N* = 19) sat for the theoretical pre-test and for theoretical post-test (*N* = 19). The comparison in the same group was to compare the effectiveness of treatment group performance after having gone through the PWMV learning process. The Descriptive statistics on theoretical pre-test and post-test is presented in S5 Table.

(S5 Table) shows the descriptive statistics on the comparison between theoretical pre-test and post-test within the same group. The analysis showed a significant improvement in the mean score in post-test with a mean score of 90.5 (±SD 7.97); *p* ≤ 0.001).

### Normality test

The Shapiro-Wilk test indicated that pre-test scores were not normally distributed (p = 0.015), while post-test scores approximated normality (p = 0.065). Because the Shapiro-Wilk test confirmed a non-normal distribution for the baseline pre-test scores (*p* = 0.015), the non-parametric Related-Samples Wilcoxon Signed-Rank Test was prioritized as the primary inferential tool to confirm a significant positive median shift (*p* = 0.001), proving the robustness of the improvement against non-normality. Therefore, the Wilcoxon Signed-Rank Test was conducted to confirm the robustness of the findings, so that it will be more specific. S6 Table; Tests of normality; theoretical pre-test and post-test, (S5 Fig); Normal Q-Q Plot of theoretical pre-test, (S6 Fig); Detrended Normal Q-Q Plot of theoretical pre-test, (S7 Fig); Normal Q-Q Plot of theoretical post-test, (S8 Fig); Detrended Normal Q-Q Plot of theoretical post-test, and (S9 Fig); Descriptives Diff_ theoretical.

### T-test

The following table shows the result for t-test for theoretical pre-test and post-test groups.

(S7 Table) presents the measurement on critical thinking skills, decision making skills and judgment with applied clinical reasoning, including analysis of patient data, clinical decision-making skills by pre-test and post-test by using descriptive analysis and paired t test analysis using IBM SPSS version 27 (IBM Corp, Armonk, NY) for teaching effectiveness results. The statistical results based on descriptive statistics in theoretical pre-test and post-test between treatment and comparison group was significant. Evidently, the post test results of student performance improved in treatment group and comparison group. The t-test results (*p* < 0.05) are shown in (S7 Table).

In treatment group (*N* = 19), there was improvement in post test results with 38 percent improvement in score. The paired-sample t-test was used to test the significance between pre-test scores and post-test score after module implementation showed significance differences. As presented in (S5 Table), the mean score between pre-test and post-test was improved from 52.89 to 90.53 percent showing that students had improved in terms of critical thinking skill, decision making skill and judgment with applied clinical reasoning, including analysis of patient data, clinical decision-making skill.

Overall, the students were more confident in these three skills when conducting the PWMV procedure by standard duration hour of practice and based on criteria weaning process procedure. The observed improvement reflects enhanced theoretical understanding and applied clinical reasoning; however, clinical outcomes were not directly measured in this study. Then, for statistical analysis, paired-sample t-test and post-test scores (*r* = .427, *p* < 0.05).

Meanwhile, the effectiveness of instructor’s practice before and after module implementation was measured on the difference between instructor on unstructured training without the module and structured training guided by PWMV pedagogical module within 6 weeks were positively increased. The results strongly showed median of differences between theoretical pre-test and post test score *p* < 0.05.

### Normality of pre-test and post test scores

Normality test was used as one of the alternative measurements to monitor significant improvement in pre- and post- theoretical score from Kolmogorov-Smirnov and Shapiro-Wilk. This Kolmogorov-Smirnov statistic is based on the largest vertical difference between hypothesis and empirical distribution of this study. However, a few common normality test procedures are available on statistical that are able to present significant under certain condition or assumption and produce different results. (S6 Table); Tests of normality; theoretical pre-test and post-test.

## Discussion

### Instructor and CICU nurse perception toward pedagogical module WPMV implementation

The module curriculum components of the teaching and learning, objective, content and curriculum skills is beneficial to Instructor’s and CICU nurses’ to give valuable feedback or ideas for WPMV pedagogical module improvement in future practice. Simultaneously, the practice has achieved safe practice result, in order to guarantee the future advanced nursing education in Malaysia practice attains the same level as global practice. The semi structured interview non-face to face survey was conducted with 2 instructors and 5 students involved in module implementation. thematic analysis result of reflective feedback from semi-structured interview non face to face on instructor version module and student version module based on the usability of the Weaning Process from Mechanical Ventilation (WPMV) pedagogical module for advanced nursing education.

*“Yes, all of us strongly agreed that module for instructor and module for students is really useful for teaching and learning modules of WPMV process in hardcopy to help in the teaching and learning process” [Instructor and students; 1-4]*

*“Yes, we are strongly agreed with curriculum component provided such as teaching and learning module, objectives, content, curriculum skills and values” [Instructor 1 & 2 and student 1,4 & 5; 9-23]*

*“Both of us also agreed but we as students don’t really understand in depth about curriculum but both of us agreed that all teaching and learning, objective, content and practical provide is really appropriate and effective for us to learn about WPMV [student 2 & 3; 19, 22]*

*Besides that, the module teaching and learning activities also encouraged learning in critical thinking, decision making and judgment skills through problem-based learning when they are performed to real patient scenario by daily case basis. They are found this lesson useful and benefits to their practical knowledge and skills.*

*‘Yes all of us strongly agreed and agreed that the Curriculum Assessment in the developed module can be used in the curriculum of teaching and learning WPMV in accordance with the Advanced education of nursing in cardiothoracic intensive care unit [Instructor 1 & 2 and students 1-5; 24-67]”*

*“Yes, is really useful and effective learning style through Scenario (Critical thinking, decision making and judgment skills) and reflective [Student 1-5;189-190]”*

### Discussion on challenges in pedagogical module WPMV implementation

When implementing the weaning process from mechanical ventilation (WPMV) pedagogical module within the cardiothoracic intensive care unit (CICU), three primary institutional challenges emerged that require consideration for future curriculum optimization. Identifying these influencing factors is essential to address the systemic limitations observed during the study. First, while the clinical instructors functioned as subject matter experts with extensive domain knowledge, they frequently lacked formal training in foundational pedagogical principles and instructional design. Second, a pervasive global shortage of senior nursing personnel relative to junior staff created a high-acuity workload, which severely limited the capacity and willingness of senior clinicians to engage in formal teaching and learning activities. Lastly, operational constraints arose from coordinating intensive clinical training alongside the rigid shift schedules of actively working postgraduate students within the active CICU practice environment

### Instructor challenges

The primary operational hurdle encountered by clinical instructors was a lack of formal pedagogical training. Functioning primarily as clinical practitioners rather than career academicians, this instructional gap caused minor delays. Although highly proficient subject matter experts, they had no prior involvement in structured curricular designs or accreditation programs due to their baseline focus on bedside teaching within the National Heart Institute (IJN) cardiothoracic intensive care unit (CICU). Consequently, their familiarity with foundational pedagogical principles remained minimal. Core responsibilities prioritized clinical services and patient management, supplemented by roles as departmental education coordinators. Nevertheless, each was an organizationally certified clinical mentor officially sanctioned to execute the module. A compounding challenge was the critical shortage of senior nursing personnel. To remedy these global pandemic-era staffing deficits, future iterations should engage three to four instructors to comply with the Nursing Board Malaysia’s mandated 1:15 educator-to-student ratio. Despite these structural constraints, strong collaborative teamwork within the IJN CICU enabled successful module completion within the designated six-week timeline

### Student challenges

Subsequently, three categories of student-related challenges emerged during module implementation that warrant future curricular adjustment. First, participating nurses experienced acute environmental stressors, including high patient volumes, significant clinical work overload, and systemic tension. This high-acuity baseline was compounded by chronic nursing shortages that directly impeded the timeline of instructional activities and evaluations. Second, operational complexities arose from coordinating intensive training alongside the rigid shift schedules of actively working postgraduate students within the active CICU practice environment. Lastly, baseline knowledge deficits heightened the early learning curve regarding the mechanical ventilation weaning process. Overcoming these entry-level gaps was critical, as competent cognitive processing during weaning procedures prevents clinical delays that could precipitate weaning failure. Throughout the program, students were required to synthesize complex physiological parameters to demonstrate safe, effective weaning performance

### Other challenges related during implementation

A major challenge stemmed from conducting the module implementation during the critical COVID-19 pandemic, which imposed global constraints that required adaptation to an unprecedented clinical environment. This process faced delays due to strict adherence to new pandemic-related standard operating procedures. Furthermore, serial cross-transmissions infected multiple personnel, leaving students with severe difficulties when trying to select non-infectious, stable patient cases for their mandatory logbook reviews. Extensive quarantine mandates and contact limitations frequently interrupted instructional progress

### Pedagogical module WPMV effectiveness

Module effectiveness was evaluated by comparing theoretical pre-test and post-test trends within the intervention group. The analysis revealed a statistically significant improvement in mean scores, which increased from 52.89% (±SD 10.18) at baseline to 90.53% (±SD 7.97) post-intervention (p ≤ 0.001). These metrics demonstrate enhanced theoretical knowledge performance and domain-specific applied clinical reasoning capacities following module completion. Descriptive performance benchmarks also verified that lower-performing participants successfully reached the mandated institutional competency thresholds, generating increased confidence during simulated clinical procedures. Concurrently, the operational impact on instructor practice was evaluated over the six-week implementation phase. Transitioning from unstructured work-based training to the structured pedagogical module yielded an evident positive shift, with a non-parametric hypothesis test summary confirming a significant positive median difference between assessment phases (*p* < 0.05).

### Discussion on evaluation and implementation

This section evaluates the final six-week implementation phase of the weaning process from mechanical ventilation (WPMV) pedagogical module. The evaluation involved an intervention cohort (*n* = 19) and a comparison benchmark cohort (*n* = 5). Participants in the intervention group were briefed on the module’s core learning objectives, educational content, instructional strategies, and multi-tiered evaluation metrics. Although operational challenges emerged, the implementation progressed smoothly and concluded in alignment with the instructional plan [20].

The pedagogical delivery was structured around Gagné’s Nine Events of Instruction. During the initial two weeks, activities focused on gaining trainee attention, establishing clear expectations by detailing module objectives, and stimulating the retrieval of prior baseline knowledge. By the second week, the instructor introduced selective clinical stimuli through intensive practical attachments, offering systematic learning guidance and eliciting active student performance via continuous clinical assessments. During the final two weeks, instructors provided targeted, reinforcement-based feedback, allowing participants to engage in meaningful self-reflection and initiate independent strategies for self-improvement. The concluding week involved comprehensive summative evaluations, consisting of theoretical post-tests alongside diverse practical assessments, including logbook reviews, clinical vivas, and formal case presentations.

Integrating Gagné’s framework yielded excellent student performance and heightened instructional motivation. Post-test metrics confirmed the module’s baseline efficacy in both theoretical and practical domains. Instructors noted that students demonstrated advanced cognitive capacities, providing strong remedial feedback during oral questioning and successfully applying complex concepts to reinforce their clinical skills. This developmental growth was starkly apparent in bedside applications, specifically regarding autonomous execution of the weaning algorithm, precise calculation of the rapid shallow breathing index (RSBI), and the management of Spontaneous Breathing Trials (SBT). Ultimately, these outcomes reinforce that effective advanced training relies on structured lesson planning that offers a holistic view of WPMV protocols [21].

### Reflective learning and systemic challenges

The incorporation of Johns’ Model of Reflection further supported student knowledge optimization. This reflective approach required nurses to critically analyze their clinical actions before, during, and after practical assignments within the cardiothoracic intensive care unit (CICU). Reflective learning allowed trainees to contrast historical habits with current evidence-based practices, translating classroom theory into structured clinical mastery. Daily reflection is vital in high-acuity environments; encouraging reflective dialogue during logbook reviews empowered students to accurately evaluate their own performance gaps and implement instructor feedback.

The WPMV module directly addresses an essential curriculum gap, as no standardized, subspecialty pedagogical frameworks previously existed within the Malaysian advanced nursing context. However, three operational challenges must be acknowledged to guide future iterations. First, clinical instructors frequently exhibited baseline pedagogical skill deficits. While functioning as highly proficient subject matter experts, they lacked formal educator training. Second, severe institutional staffing deficits restricted the availability of senior personnel to support the training program. Finally, the study was conducted during the COVID-19 pandemic. Restrictive standard operating procedures (SOPs), extensive staff quarantines, and cross-transmission risks disrupted clinical schedules and complicated logbook case selection. Coordinating specialized training around the rigid shift patterns of working clinical students presented a secondary logistical hurdle.

Despite these acute pandemic-era constraints, the strong collaborative culture within the institute fostered high instructor enthusiasm, leading to a successful implementation within the designated six-week timeline. Minor structural limitations can be resolved through targeted future adaptations. Ultimately, these findings demonstrate that structured pedagogical interventions successfully optimize theoretical knowledge performance and applied clinical reasoning among critical care nurses. The very large educational effect size highlights a meaningful clinical impact, strongly justifying the formal integration of structured instructional designs in advanced nursing curricula.

### Limitation

A primary limitation of this study is the relatively small sample size, consisting of 24 CICU nurses. While these participants represent a critical specialized cadre within the National Heart Institute (IJN), the total N = 24 (comprising a treatment group of n = 19 and a comparison group of n = 5) restricts the overall statistical power of the findings. This small cohort size was a result of the specialized nature of cardiothoracic intensive care nursing and the operational constraints of conducting research within a single high-acuity center. Consequently, while the results demonstrate significant internal effectiveness, they should be interpreted with caution regarding broader clinical applications.

The study’s generalizability is further limited by its reliance on a single institution and a non-randomized, purposive sampling method. Since participants were allocated to the intervention cohort based on pre-test scores falling beneath the 70% institutional passing standard, the mathematical risk of a regression-to-the-mean (RTM) effect must be transparently acknowledged as a design limitation. Statistical theory dictates that selecting extreme lower-performing individuals can result in a natural upward shift during subsequent testing. However, the exceptional magnitude of the score improvement captured within our treatment cohort surging by a mean difference of 37.63 percentage points (from 52.89% to 90.53%, *p* < 0.001) vastly outstrips typical statistical RTM fluctuations. Backed by a high *t*-s*t*atistic (*t* (18) = 14.30) and a profound Cohen’s *d* effec*t* size of 3.28, these findings indicate true, systematic educational mastery rather than a statistical artifact.

The small proficient cohort (*n* = 5) was strictly implemented not for inferential comparison, but as an absolute ‘expert performance benchmark’ to verify that the underperforming group successfully reached the unit’s required level of safe, autonomous practice. The unique organizational culture, instructor-to-student ratios, and existing clinical protocols at IJN may have influenced the module’s success in ways that might not be replicable in general intensive care units or different hospital systems. Additionally, the lack of local and international benchmarking resources specifically for WPMV in advanced nursing education forced the study to rely on broader instructional technology and curriculum development guides. Future research should aim to validate the WPMV pedagogical module through multi-center studies with larger, randomized cohorts to establish its effectiveness across diverse clinical environments.

Operational challenges significantly impacted the implementation phase, particularly the timing of the study during the COVID-19 pandemic. Strict Standard Operating Procedures (SOPs) and high staff infection rates led to delays and forced personnel into quarantine, interrupting the educational process. These factors created difficulties for students in selecting “clean” patients for logbook cases and made it challenging to coordinate classroom and clinical sessions due to nursing shift schedules. Despite these hurdles, the high degree of cooperation from the panel of experts and the strong consensus among instructors regarding the necessity of structured WPMV training allowed the study to demonstrate the module’s potential as a crucial guiding tool for future advanced nursing education.

### Future recommendations

The development of the WPMV pedagogical module was fundamentally driven by the current and future necessity for standardized, value-based practices in advanced nursing education specialized for the Cardiothoracic Intensive Care Unit (CICU) at the National Heart Institute (IJN). By utilizing a design and development research (DDR) approach, this study establishes a structured framework intended to improve the cognitive foundations of clinical procedures, thereby preparing nurses to better recognize and mitigate potential medical complications and medico-legal ethics violations in nursing practice [6,7,22,23].

As advanced intensive technology becomes more deeply integrated into the healthcare system, the expectations for nursing roles are automatically shifting; nurses must now act as highly competent care providers who utilize technology to maintain quality and patient safety [24]. This technological progression serves as a vital tool to assist CICU nurses in complex clinical decision-making and patient management. Because the job scope in the CICU remains exciting and demanding, the implementation of such specialized modules increases the value of nursing experience both in Malaysia and internationally. Evidence from existing literature supports the feasibility and safety of nurse-led weaning protocols, suggesting that clinical nurse specialists can significantly reduce postoperative complications and improve patient management in intermediate care environments [25].

Moving forward, the CICU, the National Heart Institute, and the Nursing Board Malaysia (NBM) should increase the complexity of the WPMV training program to better prepare clinicians for 21st-century healthcare challenges. Future curricula must prioritize a robust foundation in applied clinical reasoning and advanced decision-making to foster greater nursing autonomy. To safeguard instructional quality, the NBM curriculum development unit must stringently monitor advanced courses, adhering to regulations mandated by the Ministry of Higher Education and the Malaysian Qualifications Agency. Such oversight remains crucial to prevent clinical incompetence, thereby mitigating risks of negligence, malpractice, and elevated mortality. Furthermore, the profession should be supported in expanding clinical roles within legal boundaries, conforming to the NBM Code of Professional Conduct’s mandate of 10 annual continuing education hours to maintain competence [26,27].

This study provides essential empirical evidence regarding specific learning objectives and evaluative processes that can benefit the CICU long-term. The findings from the needs assessment and expert consensus offer a robust foundation for policy makers in the Ministry of Higher Education (MOHE) to reference when designing future specialized nursing curricula [28]. Additionally, the methodologies employed during the design phase, such as the Fuzzy Delphi technique, should be expanded within the organization’s research departments to focus on institutional improvement beyond standard clinical trials. The experts involved in this study should be utilized as lead trainers for future implementations, and their contributions should be recognized as yearly key performance indicators (KPIs) to motivate staff and foster a culture of educational excellence within the CICU [22].

## Conclusion

The development of the WPMV pedagogical module, grounded in a comprehensive needs analysis, provides a structured framework that successfully bridges the gap between theoretical knowledge and clinical practice in the CICU. By simulating actual work environments through established instructional design, the module equips nurses with the competencies necessary to meet global standards of high-quality care [29]. This study demonstrates that the structured intervention is highly effective, yielding a statistically significant improvement in theoretical scores from 52.9% to 90.5% (p < 0.001), thereby enhancing critical thinking and clinical judgment in high-acuity settings.

Beyond immediate educational gains, the implementation of this standardized module serves as a vital pedagogical foundation that is conceptually anticipated to support the long-term reduction of medico-legal risks and clinical complications associated with mechanical ventilation weaning [6,7]. Furthermore, these findings support the evolving role of Clinical Nurse Specialists (CNS) within the Malaysian cardiovascular team, fostering greater professional autonomy and service quality. Overall, the WPMV module establishes a robust pedagogical benchmark for advanced nursing education, ensuring safer patient outcomes and higher standards of professional excellence in cardiothoracic care.

This study demonstrates that the WPMV pedagogical module is effective in improving theoretical knowledge and applied clinical reasoning among CICU nurses. While the findings support the integration of structured educational interventions in advanced nursing education, further research is required to evaluate long-term clinical outcomes and generalizability across settings.

## Supporting information

S1 File Raw data supporting the findings of this study. (DOCX)

S1 Fig Theoretical framework for developing a weaning process from mechanical ventilation WPMV module for advanced nursing education. (DOCX)

S2 Fig Taba guide – eight-steps sequence in the process of developing and design curriculum. (DOCX)

S3 Fig Others teaching and learning materials. (DOCX)

S4 Fig Module implementation. (DOCX)

S5 Fig Normal Q-Q Plot of theoretical pre-test. (DOCX)

S6 Fig Detrended Normal Q-Q Plot of theoretical pre-test. (DOCX)

S7 Fig Normal Q-Q Plot of theoretical post test. (DOCX)

S8 Fig Detrended Normal Q-Q Plot of theoretical post test. (DOCX)

S9 Fig Descriptive Diff_ theoretical. (DOCX)

S1 Table Gagne’s 9 events of instruction. (DOCX)

S2 Table Distribution of respondents based on the cut-point (Score) 70 marks of 24 students. (DOCX)

S3 Table Control and treatment groups in design of development a WPMV module. (DOCX)

S4 Table Case processing summaries on pre-test and post test. (DOCX)

S5 Table Descriptives statistics on theoretical pre-test and post test. (DOCX)

S6 Table Tests of normality; theoretical pre-test and post test. (DOCX)

S7 Table Paired sample statistics, paired sample correlation, paired sample test, paired sample effects sizes and hypothesis test summary for T-test theoretical pre-test and post test. (DOCX)
