# Supplementary material for: Effectiveness of a pedagogical module for the process of weaning from mechanical ventilation in advanced nursing education
Source: PLoS One. 2026 Jun 29;21(6):e0332792. doi: 10.1371/journal.pone.0332792 (PMC13313338; doi:10.1371/journal.pone.0332792)
Supplement: S1 File — (DOCX) [file pone.0332792.s001.docx]

| 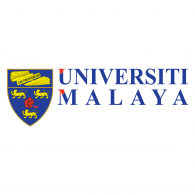 | **RESULTS TREATMENT GROUP**  **Research Project Implementation**  **[1^st^ March 2021 – 9^th^ April 2021]** |
| --- | --- |

| **Module Title** | **Development of a Weaning Process from Mechanical Ventilation (WPMV) Pedagogical Module for Advanced Nursing Education** |
| --- | --- |

| **SN** | **Name** | **Staff ID** | **21/2/21** | **5/3/2021** | **Pre-test - Post-test**  **(%)** | **Total Mark**  **(%)** | **25/3/2021 & 26/3/2021** | **7/4/2021** | **Total Mark**  **(%)** | **Grand Total** |
| --- | --- | --- | --- | --- | --- | --- | --- | --- | --- | --- |
|  |  |  | **Theoretical** | |  |  | **Practical** | |  |  |
|  |  |  | **Pre-Test (%)** | **Post- Test (%)** |  |  | **Case Study Presentation (Viva) (%)** | **Log Book (%)** |  |  |
| 1 | Azma Hazwani Salahuddin | 4277 | 25 | 90 | 65 | 90 | 76 | 94 |  |  |
| 2 | Chitthiva A/P Prachak | 4522 | 55 | 80 | 25 | 80 | 69 | 89 |  |  |
| 3 | Fatin Hanani Binti Hashim | 4369 | 45 | 85 | 40 | 85 | 76 | 93 |  |  |
| 4 | Fatin Nadhirah Binti Mohamad Nasi | 4458 | 60 | 95 | 35 | 95 | 80 | 88 |  |  |
| 5 | Irsyad Muhaimin Bin Mohamad | 4674 | 50 | 85 | 35 | 85 | 74 | 93 |  |  |
| 6 | Laily Azmira Binti Tarmizi | 4019 | 50 | 90 | 40 | 90 | 81 | 86 |  |  |
| 7 | Nasir Bin Badarun | 4140 | 65 |  | Transfer Department | | | | | |
| 8 | Nor Faeziah Bt Jaafar | 4286 | 60 | 75 | 15 | 75 | 68 | 90 |  |  |
| 9 | Nor Hanisah Izzati Binti Khamis | 4109 | 55 | 80 | 25 | 80 | 79 | 81 |  |  |
| 10 | Norhawadah Binti Ismail | 4710 | 50 | 100 | 50 | 100 | 83 | 89 |  |  |
| 11 | Nur Afini Binti Mohamad Naain | 4572 | 35 | 80 | 45 | 80 | 79 | 97 |  |  |
| 12 | Nur Amyra Fatin Bt Othman | 4327 | 60 | 95 | 35 | 95 | 85 | 90 |  |  |
| 13 | Nurfatin Aina Binti Mocktar | 4459 | 55 | 100 | 45 | 100 | 78 | 93 |  |  |
| 14 | Nurhabibah Binti Ab.Aziz | 4357 | 60 | 100 | 40 | 100 | 73 | 85 |  |  |
| 15 | Nurshakira Nazleen Bt Mt Nawi | 4100 | 65 | 90 | 25 | 90 | 79 | 76 |  |  |
| 16 | Nursyahidatul Aini Binti Awang Muda | 4573 | 65 | 95 | 30 | 95 | 79 | 79 |  |  |
| 17 | Nursyariena Binti Hanafi | 4711 | 45 | 90 | 45 | 90 | 79 | 88 |  |  |
| 18 | Nurul ‘Ain Binti Amirruddin | 4457 | 50 | 100 | 50 | 100 | 89 | 92 |  |  |
| 19 | Nurul Aqilah Junor | 4276 | 60 | 90 | 30 | 90 | 76 | 94 |  |  |
| 20 | Razila Bt Ramli | 4265 | 60 | 100 | 40 | 100 | 81 | 87 |  |  |

| 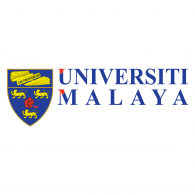 | **RESULTS CONTROL GROUP**  **Research Project Implementation**  **[1^st^ March 2021 – 9^th^ April 2021]** |
| --- | --- |

| **Module Title** | **Development of a Weaning Process from Mechanical Ventilation (WPMV) Pedagogical Module for Advanced Nursing Education** |
| --- | --- |

| **SN** | **Name** | **Staff ID** | **21/2/21** | **5/3/2021** | **Pre-test - Post-test**  **(%)** | **Total Mark**  **(%)** | **25/3/2021 & 26/3/2021** | **7/4/2021** | **Total Mark**  **(%)** | **Grand Total** |
| --- | --- | --- | --- | --- | --- | --- | --- | --- | --- | --- |
|  |  |  | **Theoretical** | |  |  | **Practical** | |  |  |
|  |  |  | **Pre-Test (%)** | **Post- Test (%)** |  |  | **Case Study Presentation (Viva) (%)** | **Log Book (%)** |  |  |
| 1 | Muhammad Najir Bin Mohamaddin | 4558 | 75 | 60 | -15 | - | - | - | - | - |
| 2 | Noor Hidayah Binti Mohd Nasir | 4501 | 80 | 50 | -30 | - | - | - | - | - |
| 3 | Nursharain Binti Mohd Ariff | 4081 | 85 | 45 | -40 | - | - | - | - | - |
| 4 | Mimi Nor Asimah Bt Setapani | 4302 | 70 | 45 | -25 | - | - | - | - | - |
| 5 | Lim Hui Xian | 3980 | 85 | 65 | -20 | - | - | - | - | - |
