# Supplementary material for: Effectiveness of a pedagogical module for the process of weaning from mechanical ventilation in advanced nursing education
Source: PLoS One. 2026 Jun 29;21(6):e0332792. doi: 10.1371/journal.pone.0332792 (PMC13313338; doi:10.1371/journal.pone.0332792)
Supplement: S1 Fig — (DOCX) [file pone.0332792.s002.docx]

**S1 Fig. Theoretical Framework for developing a weaning process from mechanical ventilation WPMV module for Advanced Nursing Education**

~~
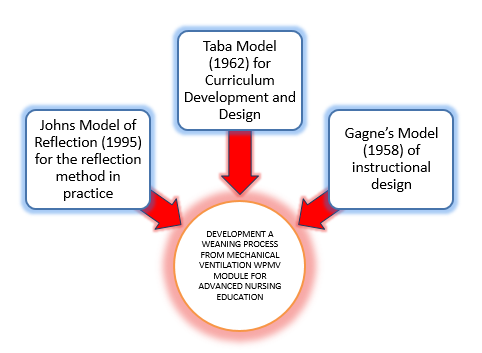
~~
