# Supplementary figures and images for: Effectiveness of a pedagogical module for the process of weaning from mechanical ventilation in advanced nursing education
Source: PLoS One. 2026 Jun 29;21(6):e0332792. doi: 10.1371/journal.pone.0332792 (PMC13313338; doi:10.1371/journal.pone.0332792)

**S2 Fig . Taba guide - eight-steps sequence in the process of developing and design curriculum**


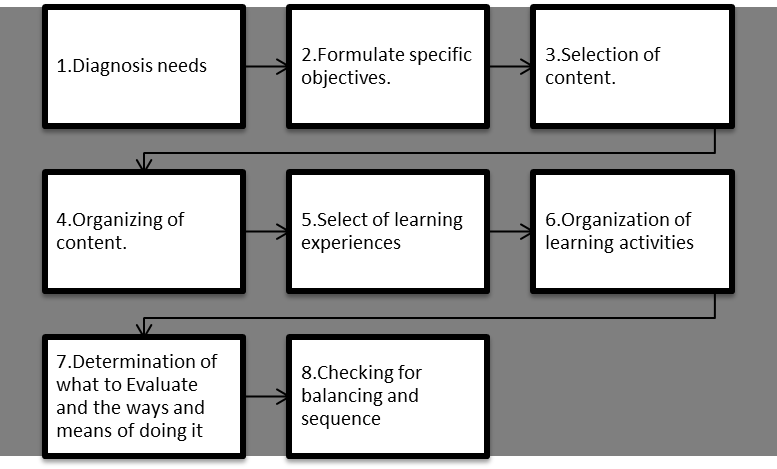

Supplement: S2 Fig — (DOCX) [file pone.0332792.s003.docx]

**S3 Fig. Others teaching and learning materials.**


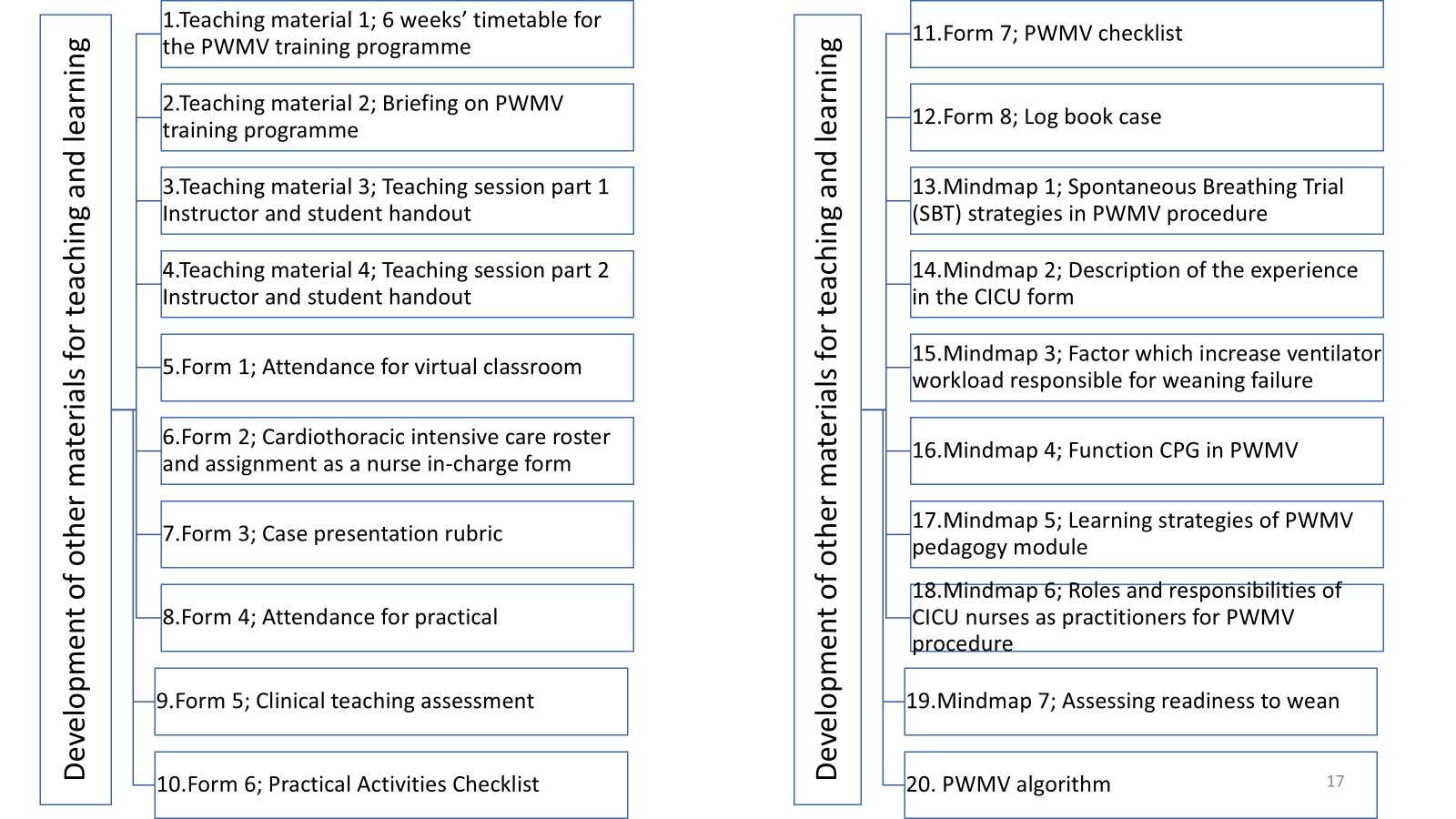

Supplement: S3 Fig — (DOCX) [file pone.0332792.s004.docx]

**S4 Fig. Module Implementation.**


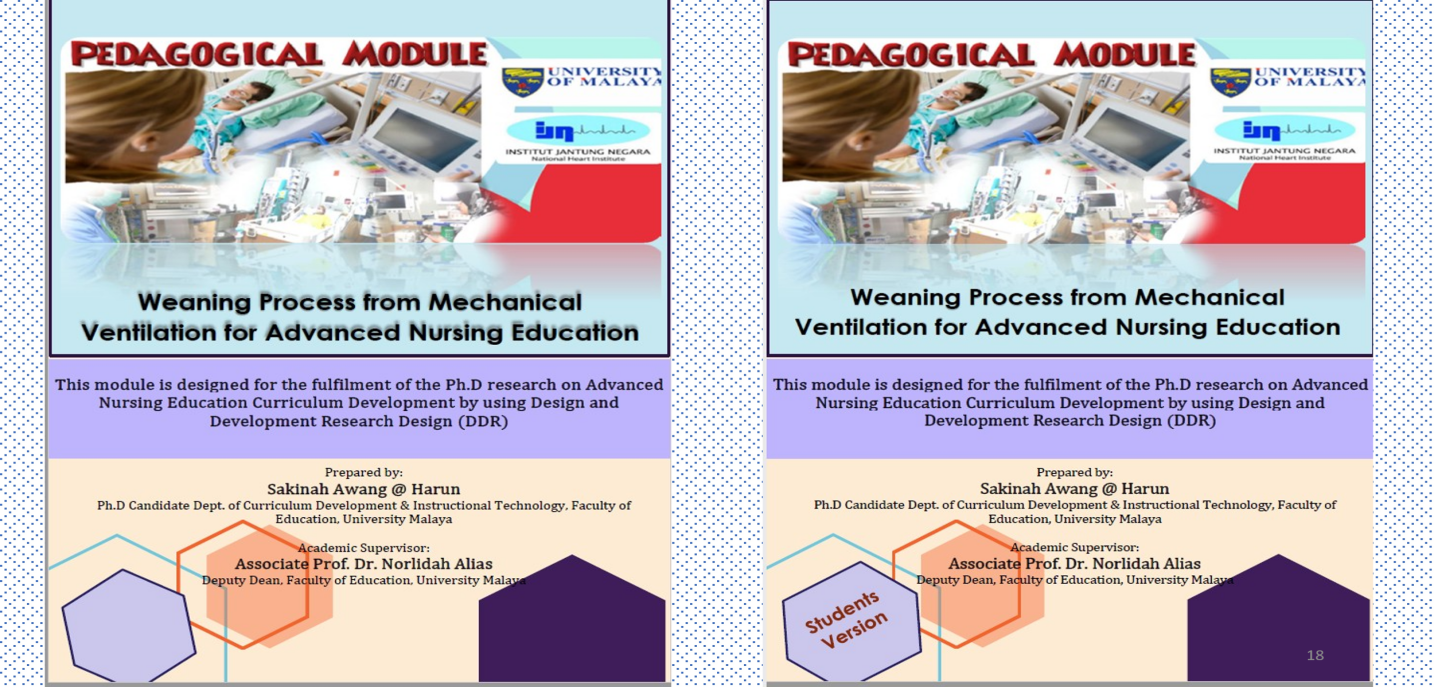


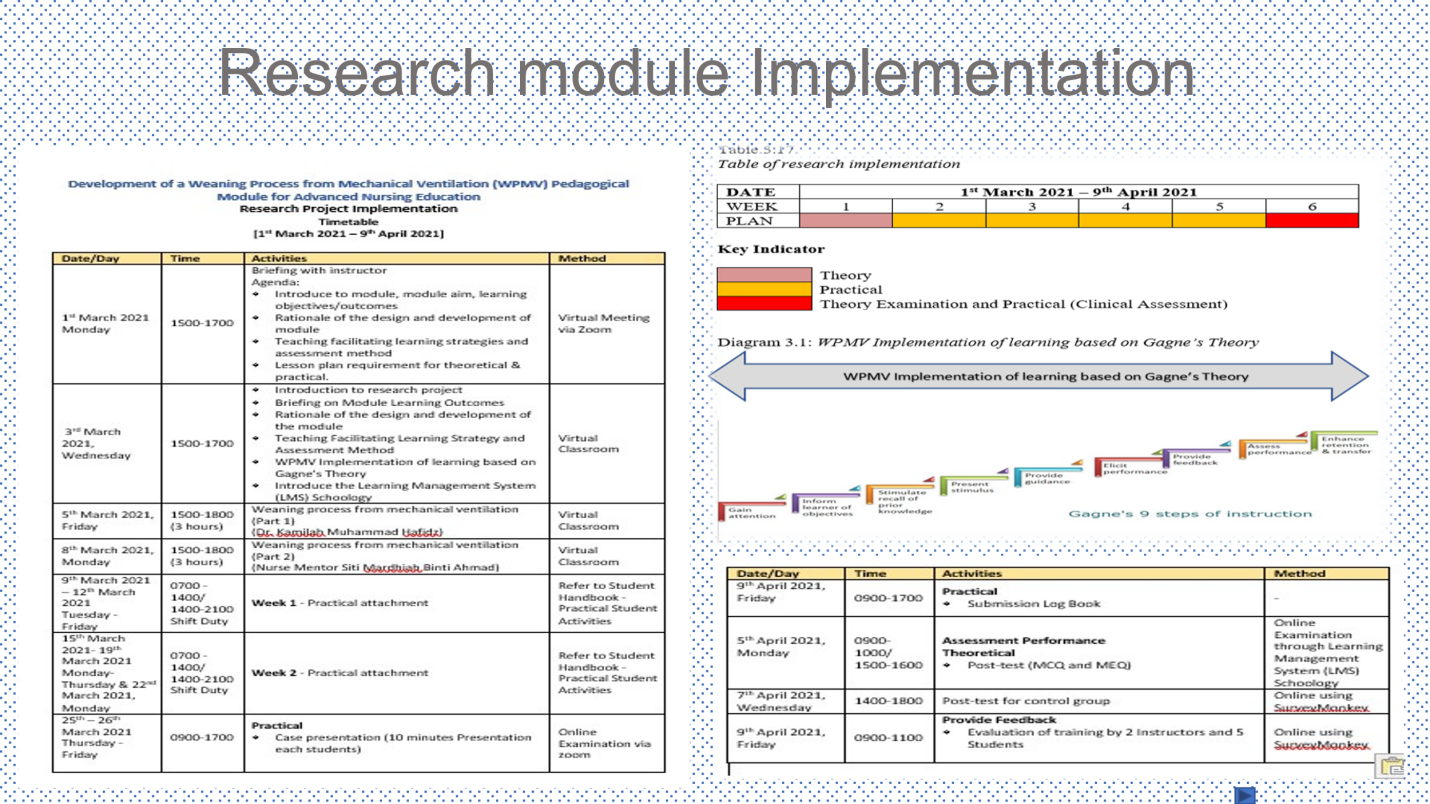

Supplement: S4 Fig — (DOCX) [file pone.0332792.s005.docx]

**S5 Fig. Normal Q-Q Plot of theoretical pre-test.**


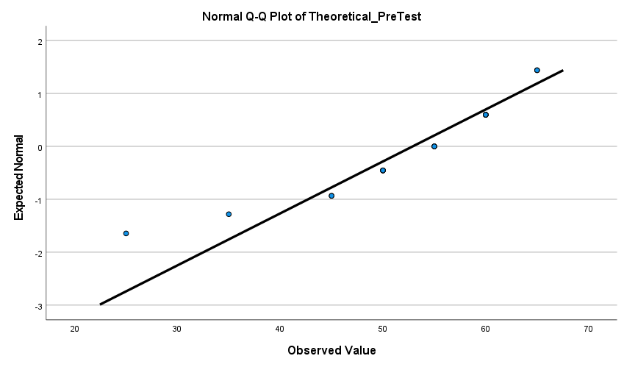

Supplement: S5 Fig — (DOCX) [file pone.0332792.s006.docx]

**S6 Fig. Detrended Normal Q-Q Plot of theoretical pre-test**


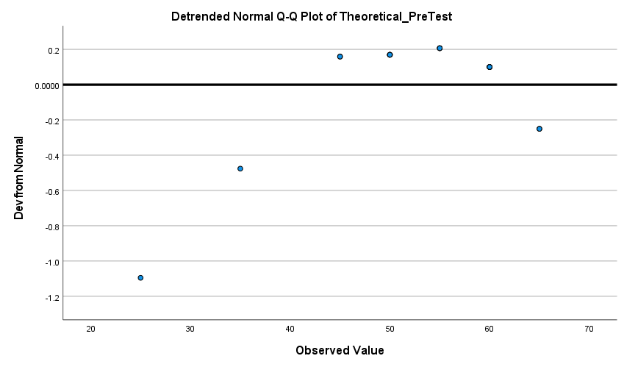

Supplement: S6 Fig — (DOCX) [file pone.0332792.s007.docx]
