# Supplementary material for: Effectiveness of a pedagogical module for the process of weaning from mechanical ventilation in advanced nursing education
Source: PLoS One. 2026 Jun 29;21(6):e0332792. doi: 10.1371/journal.pone.0332792 (PMC13313338; doi:10.1371/journal.pone.0332792)
Supplement: S1 Table — (DOCX) [file pone.0332792.s011.docx]

**S1 Table. Gagne’s 9 Events of Instruction**

| No | Gagné’s Nine events of instruction | Methods |
| --- | --- | --- |
| 1 | **Gain attention of the student (reception)**  Ensure the students are ready to learn and participate in activities by presenting a stimulus to gain their attention. | **Methods for gaining student attention include:**   - Stimulate students with novelty, uncertainty and surprise - Pose thought-provoking questions - Have students pose questions to be answered by other students |
| 2 | **Inform students of the objectives (expectancy)**  Inform students of the objectives or outcomes to help them comprehend what they must learn during the course. Provide objectives before instruction begins. | - Methods of stating the outcomes include: - Describe required performance - Describe criteria for standard performance - Learner establishes criteria for standard performance |
| 3 | **Stimulate recall of prior learning (retrieval)**  Help students make sense of new information by relating it to something they already know or something they have already experienced | **Methods for stimulating recall include:**   - Ask questions about past experiences - Ask students about their understanding of previous concepts |
| 4 | **Present the content (selective perception)**  Use strategies to present and cue lesson content to provide more effective, efficient instruction. Organise and chunk content in a meaningful way. Provide explanations after demonstrations. | **Ways to present and cue lesson content include:**   - Provide examples - Present multiple versions of the same content, e.g., Video, demonstration, lecture, podcast, group work - Use a variety of media to address different learning preferences |
| 5 | **Provide learning guidance (semantic encoding)**  Advise students of strategies to aid them in learning content and of resources available. | **Methods of providing learning guidance include:**   - Provide the instructional support needed – such as scaffolds (cues, hints, prompts) which can be removed after the learner masters the task or content - Varied learning strategies – mnemonics, concept mapping, role playing, visualising - Use examples and non-examples – in addition to providing examples, use non-examples to help student’s see what not to do or the opposite of examples - Provide case studies, analogies, visual images and metaphors – case studies of real-world application, analogies for knowledge construction, visual images to make visual associations, metaphors to support learning |
| 6 | **Elicit performance (practice) (responding)**  Activate learner's processing to help them internalise new skills and knowledge and to confirm correct understanding of these concepts | **Ways to activate learner's processing include:**   - Elicit learner activities – ask deep-learning questions, refer to what students already know or have students collaborate with their peers - Elicit recall strategies – ask students to recite, revisit, or reiterate information they have learned - Facilitate learner elaborations – ask students to elaborate or explain the details and provide more complexity to their responses - Help students integrate new knowledge – provide content in a context-rich way (use real-world examples) |
| 7 | **Provide feedback** **(reinforcement)**  Provide immediate feedback on student’s’ performance to assess and facilitate learning. | **Types of feedback include:**   - Confirmatory feedback – Informs the learner they did what he or she were supposed to do - Corrective and remedial feedback – informs the learner the accuracy of their performance or response - Remedial feedback – Directs students in the right direction to find the correct answer, but does not provide the correct answer - Informative feedback – Provides information (new, different, additions, suggestions) to a learner and confirms that you have been actively listening – this information allows sharing between two people - Analytical feedback – Provides the learner with suggestions, recommendations, and information for them to correct their performance |
| 8 | **Assess performance (retrieval)**  In order to evaluate the effectiveness of the instructional events, you must test to see if the expected learning outcomes have been achieved. Performance should be based on previously stated objectives. | **Methods for testing, learning include:**   - Pretest for mastery of prerequisites - Use a pretest for endpoint knowledge or skills - Conduct a post-test to check for mastery of content or skills - Embed questions throughout instruction through oral questioning and/or quizzes - Include an objective or criterion-referenced performances which measure how well a learner has learned a topic - Identify normative-referenced performances which compares one learner to another learner |
| 9 | **Enhance retention and transfer to the job (generalisation)**  To help students develop expertise, they must internalise new knowledge. | - **Methods for helping student’s internalise new knowledge include:** - Generating examples - Create concept maps or outlines - Create job-aids, references, templates, or wizards |
