# Supplementary material for: Effectiveness of a pedagogical module for the process of weaning from mechanical ventilation in advanced nursing education
Source: PLoS One. 2026 Jun 29;21(6):e0332792. doi: 10.1371/journal.pone.0332792 (PMC13313338; doi:10.1371/journal.pone.0332792)
Supplement: S2 Table — (DOCX) [file pone.0332792.s012.docx]

**S2 Table. Distribution of respondents based on the cut-point (Score) 70 marks of 24 students**

| Distribution of participants based on the cut-point (Score) 70 marks of 24 students | | | |
| --- | --- | --- | --- |
| Student | Ability to analyse data score | Distribution | Number of Respondents |
|  |  | Group weak in data analysis | Total number of students: |
| 1 | 25 | GROUP 1 Group weak in data analysis | 19 |
| 2 | 35 |  |  |
| 3 | 45 |  |  |
| 4 | 45 |  |  |
| 5 | 50 |  |  |
| 6 | 50 |  |  |
| 7 | 50 |  |  |
| 8 | 50 |  |  |
| 9 | 55 |  |  |
| 10 | 55 |  |  |
| 11 | 55 |  |  |
| 12 | 60 |  |  |
| 13 | 60 |  |  |
| 14 | 60 |  |  |
| 15 | 60 |  |  |
| 16 | 60 |  |  |
| 17 | 65 |  |  |
| 18 | 65 |  |  |
| 19 | 65 |  |  |
| 20 | 70 | **Cut of point (Score) = 70** | **Total Number of students:** |
| 21 | 75 | Group 2 - Skilled in data Analysis | 5 |
| 22 | 80 |  |  |
| 23 | 85 |  |  |
| 24 | 85 |  |  |
