# Supplementary material for: Effectiveness of a pedagogical module for the process of weaning from mechanical ventilation in advanced nursing education
Source: PLoS One. 2026 Jun 29;21(6):e0332792. doi: 10.1371/journal.pone.0332792 (PMC13313338; doi:10.1371/journal.pone.0332792)
Supplement: S3 Table — (DOCX) [file pone.0332792.s013.docx]

**S3 Table. Comparison and Treatment Groups in the Design and Development of a WPMV Module.**

| Group |  |  |  |  |
| --- | --- | --- | --- | --- |
| Treatment Group | N1  (19 students) | M1  (19 students) | X  (19 students) | M3  (19 students) |
| Comparison group | N2  (5 students) | M2  (5 students) | X  (5 students) | M3  (5 students) |

Keys:

| N1 | = | Group of respondent’s students weak in data analysis same academic education background with bachelor with three year or less than three years working experience in CICU |
| --- | --- | --- |
| N2 | = | Group of respondent’s students skilled in data analysis same academic education background with bachelor with three year or less than three years working experience in CICU |
| X | = | Treatment of student weak in data analysis |
