# Supplementary material for: Effectiveness of a pedagogical module for the process of weaning from mechanical ventilation in advanced nursing education
Source: PLoS One. 2026 Jun 29;21(6):e0332792. doi: 10.1371/journal.pone.0332792 (PMC13313338; doi:10.1371/journal.pone.0332792)
Supplement: S4 Table — (DOCX) [file pone.0332792.s014.docx]

**S4 Table. Case processing summaries on pre-test and post test**

| **Case Processing Summary** | | | | | | | | |
| --- | --- | --- | --- | --- | --- | --- | --- | --- |
|  | Cases | | | | | | | |
|  | Valid | | Missing | | | Total | | |
|  | N | Percent | | N | Percent | | N | Percent |
| Theoretical Pretest | 19 | 100.0% | | 0 | 0.0% | | 19 | 100.0% |
| Theoretical Posttest | 19 | 100.0% | | 0 | 0.0% | | 19 | 100.0% |
