# Supplementary material for: Effectiveness of a pedagogical module for the process of weaning from mechanical ventilation in advanced nursing education
Source: PLoS One. 2026 Jun 29;21(6):e0332792. doi: 10.1371/journal.pone.0332792 (PMC13313338; doi:10.1371/journal.pone.0332792)
Supplement: S5 Table — (DOCX) [file pone.0332792.s015.docx]

**S5 Table. Descriptives statistics on theoretical pre-test and post test**

| **Descriptives** | | | | |
| --- | --- | --- | --- | --- |
|  | | | Statistic | Std. Error |
| Theoretical Pretest | Mean | | 52.89 | 2.336 |
|  | 95% Confidence Interval for Mean | Lower Bound | 47.99 |  |
|  |  | Upper Bound | 57.80 |  |
|  | 5% Trimmed Mean | | 53.77 |  |
|  | Median | | 55.00 |  |
|  | Variance | | 103.655 |  |
|  | Std. Deviation | | 10.181 |  |
|  | Minimum | | 25 |  |
|  | Maximum | | 65 |  |
|  | Range | | 40 |  |
|  | Interquartile Range | | 10 |  |
|  | Skewness | | -1.341 | .524 |
|  | Kurtosis | | 2.020 | 1.014 |
| Theoretical Posttest | Mean | | 90.53 | 1.830 |
|  | 95% Confidence Interval for Mean | Lower Bound | 86.68 |  |
|  |  | Upper Bound | 94.37 |  |
|  | 5% Trimmed Mean | | 90.86 |  |
|  | Median | | 90.00 |  |
|  | Variance | | 63.596 |  |
|  | Std. Deviation | | 7.975 |  |
|  | Minimum | | 75 |  |
|  | Maximum | | 100 |  |
|  | Range | | 25 |  |
|  | Interquartile Range | | 15 |  |
|  | Skewness | | -.375 | .524 |
|  | Kurtosis | | -.920 | 1.014 |
