# Supplementary material for: Effectiveness of a pedagogical module for the process of weaning from mechanical ventilation in advanced nursing education
Source: PLoS One. 2026 Jun 29;21(6):e0332792. doi: 10.1371/journal.pone.0332792 (PMC13313338; doi:10.1371/journal.pone.0332792)
Supplement: S6 Table — (DOCX) [file pone.0332792.s016.docx]

**S6 Table. Tests of normality; theoretical pre-test and post test**

| **Tests of Normality** | | | | | | | | |
| --- | --- | --- | --- | --- | --- | --- | --- | --- |
|  | Kolmogorov-Smirnov^a^ | | | Shapiro-Wilk | | | |  |
|  | Statistic | df | Sig. | Statistic | df | Sig. |  |  |
| Theoretical Pretest | .178 | 19 | .113 | .871 | 19 | .015 |  |  |
| Theoretical Posttest | .158 | 19 | .200^*^ | .907 | 19 | .065 |  |  |
