# Supplementary material for: Effectiveness of a pedagogical module for the process of weaning from mechanical ventilation in advanced nursing education
Source: PLoS One. 2026 Jun 29;21(6):e0332792. doi: 10.1371/journal.pone.0332792 (PMC13313338; doi:10.1371/journal.pone.0332792)
Supplement: S7 Table — (DOCX) [file pone.0332792.s017.docx]

**S7 Table. Paired Sample statistics, Paired Sample Correlation, paired sample Test, Paired sample effects sizes and hypothesis test summary for T-test theoretical pre-test and post test**

| **Paired Samples Statistics** | | | | | | | | | | | | | | |
| --- | --- | --- | --- | --- | --- | --- | --- | --- | --- | --- | --- | --- | --- | --- |
|  | | | Mean | | | N | | | Std. Deviation | | | Std. Error Mean | | |
| Pair 1 | Theoretical Posttest | 90.53 | | | 19 | | | 7.975 | | | 1.830 | | |  |
|  | Theoretical Pretest | 52.89 | | | 19 | | | 10.181 | | | 2.336 | | |  |
| **Paired Samples Correlations** | | | | | | | | | | | | |  |  |
|  | | | | N | | | Correlation | | | Sig. | | |  |  |
| Pair 1 | Theoretical Posttest & Theoretical Pretest | | | 19 | | | .220 | | | .366 | | |  |  |

| **Paired Samples Test** | | | | | | |
| --- | --- | --- | --- | --- | --- | --- |
|  | | Paired Differences | | | | |
|  |  | Mean | Std. Deviation | Std. Error Mean | 95% Confidence Interval of the Difference |  |
|  |  |  |  |  | Lower |  |
| Pair 1 | Theoretical Posttest - Theoretical Pretest | 37.632 | 11.471 | 2.632 | 32.103 |  |

| **Paired Samples Test** | | | | | |
| --- | --- | --- | --- | --- | --- |
|  | | Paired Differences | t | df | Sig. (2-tailed) |
|  |  | 95% Confidence Interval of the Difference |  |  |  |
|  |  | Upper |  |  |  |
| Pair 1 | Theoretical Posttest - Theoretical_PreTest | 43.160 | 14.300 | 18 | 0.001 |

| **Paired Samples Effect Sizes** | | | | | |
| --- | --- | --- | --- | --- | --- |
|  | | | Standardizer^a^ | Point Estimate | 95% Confidence Interval |
|  |  |  |  |  | Lower |
| Pair 1 | Theoretical Posttest - Theoretical_PreTest | Cohen's d | 11.471 | 3.281 | 2.120 |
|  |  | Hedges' correction | 11.717 | 3.212 | 2.075 |

| **Paired Samples Effect Sizes** | | | |
| --- | --- | --- | --- |
|  | | | 95% Confidence Interval^a^ |
|  |  |  | Upper |
| Pair 1 | Theoretical_PostTest - Theoretical_PreTest | Cohen's d | 4.427 |
|  |  | Hedges' correction | 4.334 |

| a. The denominator used in estimating the effect sizes.  Cohen's d uses the sample standard deviation of the mean difference.  Hedges' correction uses the sample standard deviation of the mean difference, plus a correction factor. |
| --- |

| **Hypothesis Test Summary** | | | | |
| --- | --- | --- | --- | --- |
|  | Null Hypothesis | Test | Sig.^a,b^ |  |
| 1 | The median of differences between Theoretical_PreTest and Theoretical_PostTest equals 0. | Related-Samples Wilcoxon Signed Rank Test | 0.001 |  |

| **Hypothesis Test Summary** | |
| --- | --- |
|  | Decision |
| 1 | Reject the null hypothesis. |

| a. The significance level is .050. |
| --- |
| b. Asymptotic significance is displayed. |
