# Supplementary material for: Effectiveness of a pedagogical module for the process of weaning from mechanical ventilation in advanced nursing education
Source: PLoS One. 2026 Jun 29;21(6):e0332792. doi: 10.1371/journal.pone.0332792 (PMC13313338; doi:10.1371/journal.pone.0332792)
Supplement: Attachment — Submitted filename: renamed_f80ce.docx [file pone.0332792.s018.docx]

**Revision of ‘PONE-D-25-48105’**

This manuscript reports the development and evaluation of a pedagogical module for the process of weaning from mechanical ventilation in advanced nursing education within a cardiothoracic intensive care unit in Malaysia. The authors assess effectiveness using pre test and post test theoretical scores and qualitative feedback from instructors and students. I have evaluated the manuscript according to the official PLOS ONE criteria.

**Criterion 1. The study presents the results of original research**

The manuscript presents original research through the design, development, and implementation of a pedagogical module for the weaning process from mechanical ventilation. Data were collected directly from CICU nurses and instructors during a six week intervention period. The study generated original quantitative data from pre test and post test theoretical assessments and qualitative data from semi structured interviews with instructors and students. The module itself is newly developed and adapted to the Malaysian advanced nursing education context, which supports the originality of the work. The results are derived from primary data collection rather than secondary sources.

**Criterion 2. Results reported have not been published elsewhere**

The manuscript does not indicate that the results have been published previously. The pedagogical module is described as newly developed, and no earlier publications using the same dataset are cited. The data appear to originate exclusively from this single implementation involving 24 CICU nurses and two instructors. There is no evidence of duplication or reuse of previously published results.

**Criterion 3. Experiments, statistics, and analyses are performed to a high standard and are described in sufficient detail**

The study uses a quasi experimental regression discontinuity design to evaluate the effectiveness of the pedagogical module. Participants were divided into treatment and control groups based on a pre test cut off score of 70, distinguishing nurses weak in data analysis from those skilled in data analysis. The treatment group consisted of 19 participants and the control group of five participants. The intervention lasted six weeks and included theoretical teaching, clinical practice, and reflective learning guided by the module.

Statistical analyses were conducted using SPSS version 27 and included paired sample t tests, descriptive statistics, and normality tests using Kolmogorov Smirnov and Shapiro Wilk procedures. The manuscript reports an increase in mean theoretical test scores from 52.9 percent in the pre test to 90.5 percent in the post test, with p values reported as less than or equal to 0.001. These analyses are appropriate for within group comparisons and are described with sufficient procedural detail to allow replication.

However, the control group size is very small, which limits the robustness of comparative interpretation. The manuscript states that the cut off score of 70 was determined by the researcher, but no empirical or theoretical justification for this threshold is provided. Effect sizes are mentioned but not consistently reported or interpreted. Confidence intervals are shown in some tables but are not discussed in the text. The manuscript would benefit from a clearer explanation of assumptions associated with the regression discontinuity design and from a more explicit discussion of potential confounding factors such as prior experience and workload in the CICU.

**Criterion 4. Conclusions are supported by the data and presented appropriately**

The conclusions that the pedagogical module improved knowledge, critical thinking, decision making, and judgment skills are supported by the significant increase in post test scores and by qualitative feedback from instructors and students. The reported improvement in theoretical performance aligns with the stated research objectives.

However, the manuscript concludes that the module contributes to reducing complications and medico legal ethics violations in nursing practice. These claims are not directly supported by the data presented, as no patient outcomes, complication rates, or legal indicators were measured. The conclusions would be more appropriately limited to educational outcomes rather than extended to clinical and legal effects. The main conclusion regarding effectiveness in teaching and learning is justified by the data, but broader claims exceed the evidence provided.

**Criterion 5. The article is intelligible and written in standard English**

The manuscript is intelligible and the overall purpose and findings can be understood. Nevertheless, the quality of written English requires substantial improvement. There are frequent grammatical errors, awkward phrasing, and repetition throughout the text. Key constructs such as critical thinking, decision making, and judgment skills are not clearly defined and are sometimes used interchangeably. Several sections of the Discussion repeat similar descriptive statements rather than providing analytical interpretation. Professional language editing is strongly recommended to improve clarity, coherence, and scientific style.

**Criterion 6. The research meets ethical standards and is ethically sound**

The study reports that ethical approval was obtained from Institut Jantung Negara with a specified approval number. Written informed consent was obtained from all participants. The research involved only staff participants and did not include patients or animal subjects. The manuscript provides adequate information regarding ethical oversight and participant consent. These elements indicate that the research complies with accepted ethical standards for human subject research.

**Criterion 7. The article adheres to reporting guidelines and community data availability standards**

The manuscript includes statements on funding, competing interests, ethical approval, and data availability. The authors declare that no competing interests exist. A data availability statement indicates that data are stored securely by the authors. However, the statement does not clearly explain how external researchers may access anonymized data. PLOS ONE policy requires that data underlying the findings be fully available without restriction when possible. Greater specificity regarding data access procedures and formats would strengthen compliance with community standards. Some tables and figures would also benefit from clearer labeling and consistent formatting.

**Additional Recommendations**

The manuscript relies mainly on theoretical tests and instructor feedback to assess competence. Future development of the module could be strengthened by incorporating more structured and standardized competency assessment systems used in nursing education. Evidence from recent work (DOI: 10.1016/j.teln.2025.08.024) highlights the value of structured competency evaluation frameworks with defined criteria and scenario based assessment for measuring clinical judgment and professional competence. Integrating similar structured evaluation strategies could improve the validity and comparability of outcome measures in future studies.

Clear operational definitions of critical thinking, decision making, and judgment skills should be provided, together with explicit descriptions of how these constructs were measured in the theoretical tests and clinical logbook assessments. The Discussion section should focus more on interpreting the findings in relation to existing literature on nurse education and mechanical ventilation weaning rather than repeating methodological descriptions. Limitations should be stated more explicitly, particularly the small sample size, the single center design, the lack of randomization, and the short duration of follow up. Future research should consider larger multi center studies and include objective clinical performance indicators to evaluate long term impact.

**Overall Asessment**

For all these reasons, substantial revision is necessary to improve methodological transparency, align conclusions with the data, strengthen reporting compliance, and enhance linguistic and structural clarity. With these major revisions, the manuscript could make a meaningful contribution to research on pedagogical interventions in advanced nursing education.
