# Supplementary material for: Effectiveness of a pedagogical module for the process of weaning from mechanical ventilation in advanced nursing education
Source: PLoS One. 2026 Jun 29;21(6):e0332792. doi: 10.1371/journal.pone.0332792 (PMC13313338; doi:10.1371/journal.pone.0332792)
Supplement: Attachment — Submitted filename: Response to Reviewers_PONE-D-25-48105.docx [file pone.0332792.s019.docx]

Date: March 12, 2026

**To: The Editor, PLOS ONE**

**Subject**: Resubmission of Manuscript [PONE-D-25-48105]

**Title**: Effectiveness of a Pedagogical Module for The Process of Weaning from Mechanical Ventilation in Advanced Nursing Education"

**Dear Editor and Reviewers**,

We would like to express our sincere gratitude for the constructive feedback provided on our manuscript. We have performed a major revision to address each of the technical and structural concerns raised. Below is our point-by-point response to the reviewers' comments:

**Reviewer #2 Comments:**

Methodological Misrepresentation (RDD):

Comment: The manuscript repeatedly claims the use of a regression discontinuity design (RDD) without meeting methodological criteria.

**Response**: We agree and have removed all references to RDD. The study is now explicitly defined as a quasi-experimental, non-randomized pre-post-test design.

Statistical Reporting (*p* = .000):

Comment: Replace all instances of "*p* = .000" with *p* < 0.001.

**Response:** All p-values have been updated to *p* < 0.001 throughout the manuscript to maintain scientific accuracy.

Sample Size and Generalizability:

Comment: The sample size (*N* = 24) is small and restricts generalizability.

**Response:** We have expanded the Limitation section to acknowledge the small sample size (*N*=24) and have moderated our claims regarding the generalizability of the results beyond the specialized context of the National Heart Institute (IJN).

Author Summary Biography:

Comment: The Author Summary reads as a professional biography rather than a scientific summary.

**Response:** We have completely rewritten the Author Summary to focus strictly on the research rationale, methodology, and key findings.

Reviewer #3 Comments:

Normality Assumption (Shapiro-Wilk):

Comment: The Shapiro-Wilk test (pre-test) shows *p*=0.015, contradicting the assumption of normality.

**Response:** We have corrected this in the text. To ensure statistical rigor for the non-normal pre-test distribution, we have supplemented our analysis with the Wilcoxon Signed-Rank Test (*Z* = -3.825, *p* < 0.001), confirming the significance of the improvement.

Pedagogical Framework:

Comment: Add why the design is suitable and how it aligns with literature.

**Response:** We have clarified the use of the Taba, Gagné, and Johns models within the Methodology section and explained their alignment with instructional technology standards.

Attach the Revision Matric PONE-D-25-48105 for your perusal. We believe the manuscript now meets the high standards for technical soundness and clarity required by PLOS ONE.

Sincerely,

Dr. Sakinah Awang @ Harun

Corresponding Author

University of Malaya, Malaysia

**Revision Matric PONE-D-25-48105**

| No. | Reviewer / Editor Comment | Author Amendment / Action Taken | Manuscript Location |
| --- | --- | --- | --- |
| 1 | Methodological Labelling: Reviewer 2 noted the study was incorrectly labelled as "Regression Discontinuity Design". | Removed all references to RDD. The study is now correctly defined as a quasi-experimental, non-randomized pre-post design. | Abstract, Methodology (Page 1, 8) |
| 2 | Statistical Accuracy: Reviewer 2 stated that *p* = .000 is statistically incorrect. | Updated all p-values from *p* = .000 to *p* < 0.001 to ensure scientific rigor. | Abstract, Results (Page 1, 11) |
| 3 | Author Summary Content: Reviewer 2 requested removal of biographical details. | Replaced the professional biography with a research-focused summary highlighting study rationale, methods, and clinical outcomes. | Author Summary (Page 2) |
| 4 | Data Normality: Reviewer 3 noted the pre-test data was non-normal (*p* = 0.015). | Acknowledged the non-normal distribution and added the Wilcoxon Signed-Rank Test (*Z* = -3.825, *p* < 0.001) as a non-parametric validation. | Abstract, Methodology, Results (Page 1, 8, 11) |
| 5 | Sample Size Limitations: Reviewer 2 & 3 expressed concern over the small cohort (*N*=24). | Expanded the Limitations section to discuss the small sample size and moderated claims regarding generalizability. | Limitations (Page 21) |
| 6 | Reference Tallying: Reviewer 2 requested English editing and removal of redundancy. | Standardized the reference list to Vancouver style, removed duplicate entries, and ensured all in-text citations tally with the final 25-item list. | References (Page 24) |
