# Supplementary material for: Effectiveness of a pedagogical module for the process of weaning from mechanical ventilation in advanced nursing education
Source: PLoS One. 2026 Jun 29;21(6):e0332792. doi: 10.1371/journal.pone.0332792 (PMC13313338; doi:10.1371/journal.pone.0332792)
Supplement: Attachment — Submitted filename: renamed_c4ca4.docx [file pone.0332792.s020.docx]

**Revision of ‘PONE-D-25-48105R1’**

This manuscript presents the development and evaluation of a pedagogical module for the weaning process from mechanical ventilation in advanced nursing education within a cardiothoracic intensive care unit context. The study addresses an important topic in clinical education and provides original data derived from a structured educational intervention. The revised version shows improvement in several technical and reporting aspects. However, significant methodological and interpretative concerns remain before the manuscript can be considered for publication.

**Major Comments**

***Alignment of Conclusions with Evidence***

The conclusions extend beyond the data presented. The manuscript claims that the pedagogical module contributes to reducing clinical complications and legal risks. These outcomes were not measured in the study. The data are limited to theoretical test scores and qualitative feedback. The conclusions should be restricted to educational outcomes such as knowledge acquisition and perceived improvement in cognitive skills. Statements related to clinical outcomes and legal implications should be removed or clearly reframed as speculative.

***Study Design and Group Allocation***

Participants were assigned to groups using a cut off score of 70 determined by the researcher. This threshold remains insufficiently justified. There is no theoretical or empirical rationale provided to support this decision. This raises concerns about internal validity and potential bias in group allocation. A clearer justification is required, or the limitation should be explicitly acknowledged and discussed.

In addition, the control group consists of only five participants. This severely limits the strength of any comparative interpretation. The manuscript should address the implications of this imbalance and avoid overinterpretation of differences between groups.

***Statistical Analysis and Interpretation***

The inclusion of additional analyses such as the Wilcoxon Signed Rank Test and effect size calculations represents an improvement. However, these results are not adequately interpreted. Effect sizes are reported but not discussed in terms of magnitude or practical significance. Confidence intervals are presented in tables but are not integrated into the narrative.

The analysis remains focused on within group comparisons. The absence of robust between group analysis limits the strength of the conclusions. The manuscript would benefit from a clearer explanation of the analytical strategy and its limitations.

***Conceptual Clarity and Measurement***

Key constructs such as critical thinking, decision making, and clinical judgment are central to the study but remain insufficiently defined. The manuscript does not clearly explain how these constructs were operationalized or measured within the theoretical tests and assessments. Clear definitions and alignment between constructs and measurement tools are necessary to support the validity of the findings.

***Discussion and Interpretation***

The Discussion section remains largely descriptive and reiterates results rather than providing critical interpretation. There is limited engagement with existing literature on nursing education and mechanical ventilation training. The authors should strengthen this section by contextualizing their findings within current evidence and by discussing alternative explanations.

Limitations are not sufficiently developed. The manuscript should explicitly address the small sample size, single center design, non randomized allocation, short duration of follow up, and reliance on theoretical outcomes.

***Data Availability and Compliance***

The data availability statement remains unclear and does not fully comply with journal policy. The manuscript states that data are stored securely by the authors, which suggests restricted access. At the same time, it indicates that data are available within the manuscript and supporting files. This inconsistency should be resolved. The authors must provide a clear statement ensuring that data are accessible without restriction or justify any limitations in accordance with journal guidelines.

***Language and Presentation***

The manuscript remains intelligible but requires further language editing. Grammatical errors and awkward phrasing are still present. Professional editing is recommended to improve clarity and readability. Repetition across sections should be reduced, particularly in the Results and Discussion.

**Minor Comments**

Some terminology is used inconsistently and should be standardized throughout the manuscript. Tables and figures would benefit from clearer labeling and more concise captions. The Methods section could be streamlined to improve readability.
