# Supplementary material for: Effectiveness of a pedagogical module for the process of weaning from mechanical ventilation in advanced nursing education
Source: PLoS One. 2026 Jun 29;21(6):e0332792. doi: 10.1371/journal.pone.0332792 (PMC13313338; doi:10.1371/journal.pone.0332792)
Supplement: Attachment — Submitted filename: Response to Reviewers 3_PONE-D-25-48105.docx [file pone.0332792.s021.docx]

Date: May 6, 2026

**To: The Editor, PLOS ONE**

**Subject**: Resubmission of Manuscript [PONE-D-25-48105]

**Title**: Effectiveness of a Pedagogical Module for The Process of Weaning from Mechanical Ventilation in Advanced Nursing Education"

**Dear Editor and Reviewers**,

We would like to express our sincere gratitude for the constructive feedback provided on our manuscript. We have performed a major revision to address each of the technical and structural concerns raised. Below is our point-by-point response to the reviewers' comments:

This manuscript presents the development and evaluation of a pedagogical module for the weaning process from mechanical ventilation in advanced nursing education within a cardiothoracic intensive care unit context. The study addresses an important topic in clinical education and provides original data derived from a structured educational intervention. The revised version shows improvement in several technical and reporting aspects. However, significant methodological and interpretative concerns remain before the manuscript can be considered for publication.

**Reviewer #3:** (No Response) **ACADEMIC EDITOR COMMENTS: Please, revise this comment by reviewer 3. - All revised and amended**

| **No** | **Major Comments** | **Amendment** | **Page/Level** |
| --- | --- | --- | --- |
| **1** | The study uses a cutoff point 70 in a pretest made before the intervention, to create "weak" (n=19) and "skilful/control" (n=5) groups. Since the analyses are pre-post with paired samples, it is proposed to the authors the implementation of comparisons between both groups and adjustment by confusing. | We appreciate this suggestion. While we have included descriptive comparisons between groups, inferential adjustment for confounders was not feasible due to the small and unequal sample sizes.  We have explicitly acknowledged this limitation and clarified that the study’s primary objective is to evaluate within-group improvement following the intervention. | 12-18/ 298-408 |
| **2** | On the other hand, the authors used Kolmogorov-Smirnov and Shapiro-Wilk for the determination of normality. Being a small sample (n<50 or n<30), the test of choice would be Shapiro-Wilk for its greater power and precision. In this case, the authors confirm the normality of the sample (p>0.05), but in Table 8: normality test, it is indicated that the Shapiro-Wilk test (pretest) shows p=0.015 (not normal), which contradicts the assumption of normality and requires the use of non-parametric tests. | We thank the reviewer for identifying the contradiction in our normality reporting. Upon re-examination of Table 8, we acknowledge that the Shapiro-Wilk p-value (p=0.015) indicates a non-normal distribution for the pre-test data. Following the reviewer's recommendation, we have standardized our reporting to prioritize the Shapiro-Wilk test given our sample size (n < 50). Consequently, we have corrected the manuscript to reflect that non-parametric tests is specifically the Wilcoxon Signed-Rank Test were used for all pre-post comparisons. This change ensures our analytical strategy is statistically sound and properly aligned with the distribution of our data, thereby increasing the reliability of our conclusions | 12-18/ 298-408 |
| **3** | It is also proposed to the authors if it is possible to detail the instruments used in the training (exam specifications/skills, validity, reliability, rubrics, items, examples). | We have expanded the 'Materials and Methods' section and added a new Supporting Information file (S2 Appendix) to include the Exam Specifications. This includes a breakdown of how test items map to specific cognitive domains (Knowledge, Critical Thinking, and Decision-Making), examples of case-based items used, and the rubric employed for qualitative feedback. Furthermore, we have detailed the content validation process by the expert panel and provided the reliability indices (Shapiro-Wilk and Cronbach’s alpha) as discussed in previous points. This additional detail ensures the transparency of our measurement tools and supports the validity of our educational findings | 12-18/ 298-408 |

Sincerely,

Dr. Sakinah Awang @ Harun

Corresponding Author

University of Malaya, Malaysia
