# Supplementary material for: Effectiveness of a pedagogical module for the process of weaning from mechanical ventilation in advanced nursing education
Source: PLoS One. 2026 Jun 29;21(6):e0332792. doi: 10.1371/journal.pone.0332792 (PMC13313338; doi:10.1371/journal.pone.0332792)
Supplement: Attachment — Submitted filename: renamed_587b2.docx [file pone.0332792.s022.docx]

**Revision of ‘PONE-D-25-48105R2’**

The revised manuscript shows clear improvement compared with the previous submission. Several methodological and reporting concerns have been addressed appropriately, particularly regarding clarification of the quasi-experimental design, inclusion of additional statistical analyses, improved acknowledgment of study limitations, and a more cautious interpretation of findings. The study addresses an important topic in advanced nursing education and provides potentially valuable preliminary findings within a specialized clinical training context. However, several concerns remain partially unresolved, particularly regarding internal validity, conceptual overinterpretation of cognitive constructs, residual overstatement of clinical implications, and language quality. The manuscript is closer to publishable form, but additional moderate revision would further strengthen its methodological clarity and interpretive rigor.

**Alignment Between Conclusions and Evidence**

The revision has improved the alignment between the conclusions and the evidence presented. The abstract and conclusion now focus primarily on educational outcomes such as theoretical knowledge and applied clinical reasoning.

However, some statements within the manuscript still imply direct clinical impact that was not measured in the study. For example, references to improving patient safety, optimizing the weaning process, and reducing medico-legal risks remain present in sections such as the Author Summary and Introduction. These claims go beyond the available evidence and should either be removed or clearly framed as speculative future implications.

**Study Design and Group Allocation**

The explanation regarding the use of the 70% competency benchmark has improved, and the authors now clarify that the study should be interpreted as a quasi-experimental pilot educational evaluation rather than a randomized controlled comparison.

The manuscript also acknowledges the limitation of the small and unequal comparison group. This represents an important improvement.

Nevertheless, the methodological concern regarding group allocation remains only partially resolved. The intervention group consisted mainly of lower-performing participants, whereas the comparison group consisted of higher-performing participants at baseline. This creates a considerable risk of regression-to-the-mean effects and limits the interpretability of comparative outcomes.

In addition, the statement indicating that the cut-off score “was determined by the researcher herself” remains problematic and should be revised or more rigorously justified.

**Statistical Analysis and Interpretation**

The statistical reporting has improved substantially. The addition of Wilcoxon Signed-Rank testing, confidence intervals, and effect size estimates strengthens the analytical rigor of the study. The interpretation of effect sizes is also clearer than in the previous version.

The manuscript appropriately clarifies that between-group comparisons are descriptive rather than inferential because of the small and imbalanced sample sizes.

Some inconsistencies in the analytical presentation remain. Parametric paired t-tests continue to be emphasized despite evidence of non-normality in the pre-test scores. The statistical narrative could still be streamlined to improve coherence and reduce redundancy. Despite this, the overall concern has been addressed satisfactorily.

**Conceptual Clarity and Measurement**

The revised manuscript provides improved descriptions of applied clinical reasoning and explains that case-based scenarios were used during assessment. This clarification strengthens the conceptual framework.

However, the operationalization of constructs such as critical thinking, decision-making, and clinical judgment remains insufficiently supported. Although the terminology has been moderated compared with the previous version, the manuscript still presents these constructs as measured outcomes without presenting robust psychometric evidence demonstrating construct validity.

The manuscript would benefit from more consistent terminology emphasizing knowledge performance and applied clinical reasoning rather than validated measurement of higher-order cognitive constructs.

**Discussion and Limitations**

The Discussion section has improved considerably and now includes better engagement with relevant educational literature. The addition of a dedicated strengths and limitations subsection strengthens the manuscript and improves transparency.

The limitations related to sample size, non-randomized allocation, single-center design, and reliance on theoretical outcomes are now acknowledged more clearly.

**Data Availability**

The concerns regarding data availability appear to have been adequately resolved. The revised statement is clearer and more consistent with journal policy requirements.

**Language and Presentation**

The manuscript is more readable than the previous version. However, substantial grammatical and stylistic issues remain throughout the text. Several sentences still contain awkward phrasing, repetition, and syntactic inconsistencies that reduce readability.

Professional English-language editing remains strongly recommended before publication.
