# Supplementary material for: Effectiveness of a pedagogical module for the process of weaning from mechanical ventilation in advanced nursing education
Source: PLoS One. 2026 Jun 29;21(6):e0332792. doi: 10.1371/journal.pone.0332792 (PMC13313338; doi:10.1371/journal.pone.0332792)
Supplement: Attachment — Submitted filename: Response_to_Reviewers_3_PONE-D-25-48105_auresp_3.docx [file pone.0332792.s023.docx]

Date: May 29, 2026

**To: The Editor, PLOS ONE**

**Subject**: Resubmission of Manuscript [PONE-D-25-48105]

**Title**: Effectiveness of a Pedagogical Module for The Process of Weaning from Mechanical Ventilation in Advanced Nursing Education"

**Dear Editor and Reviewers**,

We would like to express our sincere gratitude for the constructive feedback provided on our manuscript. We have performed a major revision to address each of the technical and structural concerns raised. Below is our point-by-point response to the reviewers' comments:

This manuscript presents the development and evaluation of a pedagogical module for the weaning process from mechanical ventilation in advanced nursing education within a cardiothoracic intensive care unit context. The study addresses an important topic in clinical education and provides original data derived from a structured educational intervention. The revised version shows improvement in several technical and reporting aspects. However, significant methodological and interpretative concerns remain before the manuscript can be considered for publication.

**Reviewer #3:** (No Response) **ACADEMIC EDITOR COMMENTS: Please, revise this comment by reviewer 3. - All revised and amended**

| **No** | **Minor Comments** | **Amendment** | **Page/Level** |
| --- | --- | --- | --- |
| **1** | The authors have adequately responded to most key criticisms: The conclusions are now limited to educational results (knowledge and clinical reasoning), the study is now correctly presented as non-randomised pre-post quasi-experimental, the work is explicitly recognised as a pedagogical pilot study, includes explicit limitations (n small, a centre, non-randomised, etc.).  However, the manuscript presents methodologically relevant weaknesses, such as the design and sample size, where the "control group" remains conceptually questionable. This concept can be reformed by "comparison group" or similar.  On the other hand, drafting inconsistencies remain, since in several sections (Author summary, introduction and discussion) sentences still appear that suggest the improvement of patient safety, the reduction of complications and the clinical impact of the study, which partially contradicts the corrections already made by the authors.  It is also recommended to improve the writing of English, especially in the discussion and  Author summary.  Likewise, it may be relevant to clarify the limitations of the study in the abstract. | Amended. We completely agree with this stylistic refinement. We have gone through the entire manuscript and replaced the phrase "control group" with "comparison group" across all relevant narratives, ensuring the nomenclature accurately reflects our quasi-experimental pilot design.  Amended. We have carefully scrubbed the text to eliminate any direct assertions of direct clinical impact or active error prevention.  In the Introduction, the sentence regarding legal issues was rewritten to clarify that this is a "proactive educational strategy designed to minimize underlying factors associated with future risks" .  In the Discussion, similar corrections were applied to maintain a strict focus on classroom educational performance metrics.  Amended. A thorough proofreading and line-by-line editorial polish was completed across the text. We specifically targeted and completely removed several large blocks of text that had been accidentally duplicated back-to-back during a previous copy-paste compilation phase (specifically deleting duplicate statistical descriptions in the Methods section and repeated challenge blocks in the Discussion section) . Broken parenthetical phrasing has been fixed to ensure smooth academic readability.  Amended. We have updated the Abstract (Methods/Results/Conclusion)  We appreciate the reviewer's meticulous validation of our non-randomized quasi-experimental pilot parameters. A final targeted text search was executed, and the term "Control Group" has been completely purged from the document, including lingering table title strings within the Supporting Information listings at the very end of the text layout. The comparison cohort is now correctly and consistently identified across all sections of the submission. | 2-25/ 11 – 687  19-23  414-578  23-25  579-687 |

Sincerely,

Dr. Sakinah Awang @ Harun

Corresponding Author

University of Malaya, Malaysia
