# Supplementary material for: Effectiveness of a pedagogical module for the process of weaning from mechanical ventilation in advanced nursing education
Source: PLoS One. 2026 Jun 29;21(6):e0332792. doi: 10.1371/journal.pone.0332792 (PMC13313338; doi:10.1371/journal.pone.0332792)
Supplement: Peer review history

# Peer review: Decision Letter 0

**DOI:** 10.1371/journal.pone.0332792.r001

**Authors:** Javier Fagundo-Rivera

18 Feb 2026

PONE-D-25-48105

Effectiveness of a Pedagogical Module for The Process of Weaning from Mechanical Ventilation in Advanced Nursing Education

PLOS One

Dear Dr. AWANGHARUN,

Thank you for submitting your manuscript to PLOS ONE. After careful consideration, we feel that it has merit but does not fully meet PLOS ONE’s publication criteria as it currently stands. Therefore, we invite you to submit a revised version of the manuscript that addresses the points raised during the review process.

ACADEMIC EDITOR:

Dear Editorial Office,

Three reviewers have revised this manuscript.

Considering their comments, major revisions are required.

Please submit your revised manuscript by Apr 03 2026 11:59PM. If you will need more time than this to complete your revisions, please reply to this message or contact the journal office at plosone@plos.org. When you're ready to submit your revision, log on to https://www.editorialmanager.com/pone/ and select the 'Submissions Needing Revision' folder to locate your manuscript file.

Please include the following items when submitting your revised manuscript:

- A letter that responds to each point raised by the academic editor and reviewer(s). You should upload this letter as a separate file labeled 'Response to Reviewers'.
- A marked-up copy of your manuscript that highlights changes made to the original version. You should upload this as a separate file labeled 'Revised Manuscript with Track Changes'.
- An unmarked version of your revised paper without tracked changes. You should upload this as a separate file labeled 'Manuscript'.

If you would like to make changes to your financial disclosure, please include your updated statement in your cover letter. Guidelines for resubmitting your figure files are available below the reviewer comments at the end of this letter.

If applicable, we recommend that you deposit your laboratory protocols in protocols.io to enhance the reproducibility of your results. Protocols.io assigns your protocol its own identifier (DOI) so that it can be cited independently in the future. For instructions see: https://journals.plos.org/plosone/s/submission-guidelines#loc-laboratory-protocols. Additionally, PLOS ONE offers an option for publishing peer-reviewed Lab Protocol articles, which describe protocols hosted on protocols.io. Read more information on sharing protocols at https://plos.org/protocols?utm_medium=editorial-email&utm_source=authorletters&utm_campaign=protocols.

We look forward to receiving your revised manuscript.

Kind regards,

Javier Fagundo-Rivera, PhD

Academic Editor

PLOS One

Journal Requirements:

When submitting your revision, we need you to address these additional requirements.

1. Please ensure that your manuscript meets PLOS ONE's style requirements, including those for file naming. The PLOS ONE style templates can be found at

https://journals.plos.org/plosone/s/file?id=wjVg/PLOSOne_formatting_sample_main_body.pdf and

https://journals.plos.org/plosone/s/file?id=ba62/PLOSOne_formatting_sample_title_authors_affiliations.pdf

2. In the online submission form, you indicated that [all databased in secure with password in Author safe keeping databased record and documentation].

All PLOS journals now require all data underlying the findings described in their manuscript to be freely available to other researchers, either 1. In a public repository, 2. Within the manuscript itself, or 3. Uploaded as supplementary information.

This policy applies to all data except where public deposition would breach compliance with the protocol approved by your research ethics board. If your data cannot be made publicly available for ethical or legal reasons (e.g., public availability would compromise patient privacy), please explain your reasons on resubmission and your exemption request will be escalated for approval.

3. Please include captions for your Supporting Information files at the end of your manuscript, and update any in-text citations to match accordingly. Please see our Supporting Information guidelines for more information: http://journals.plos.org/plosone/s/supporting-information.

4. If the reviewer comments include a recommendation to cite specific previously published works, please review and evaluate these publications to determine whether they are relevant and should be cited. There is no requirement to cite these works unless the editor has indicated otherwise.

**Additional Editor Comments:**

Dear Authors,

Three reviewers have revised this manuscript.

Considering their comments, major revisions are required.

[Note: HTML markup is below. Please do not edit.]

**Reviewers' comments:**

Reviewer's Responses to Questions

**Comments to the Author**

1. Is the manuscript technically sound, and do the data support the conclusions?

The manuscript must describe a technically sound piece of scientific research with data that supports the conclusions. Experiments must have been conducted rigorously, with appropriate controls, replication, and sample sizes. The conclusions must be drawn appropriately based on the data presented.

Reviewer #1: Yes

Reviewer #2: Partly

Reviewer #3: Yes

**********

2. Has the statistical analysis been performed appropriately and rigorously?

Reviewer #1: No

Reviewer #2: No

Reviewer #3: Yes

**********

3. Have the authors made all data underlying the findings in their manuscript fully available?

The PLOS Data policy requires authors to make all data underlying the findings described in their manuscript fully available without restriction, with rare exception (please refer to the Data Availability Statement in the manuscript PDF file). The data should be provided as part of the manuscript or its supporting information, or deposited to a public repository. For example, in addition to summary statistics, the data points behind means, medians and variance measures should be available. If there are restrictions on publicly sharing data—e.g. participant privacy or use of data from a third party—those must be specified.

Reviewer #1: No

Reviewer #2: Yes

Reviewer #3: Yes

**********

4. Is the manuscript presented in an intelligible fashion and written in standard English?

PLOS ONE does not copyedit accepted manuscripts, so the language in submitted articles must be clear, correct, and unambiguous. Any typographical or grammatical errors should be corrected at revision, so please note any specific errors here.

Reviewer #1: Yes

Reviewer #2: No

Reviewer #3: Yes

**********

5. Review Comments to the Author

Please use the space provided to explain your answers to the questions above. You may also include additional comments for the author, including concerns about dual publication, research ethics, or publication ethics. (Please upload your review as an attachment if it exceeds 20,000 characters)

**Reviewer #1: SEE DOCUMENT ATTACHED**

This manuscript reports the development and evaluation of a pedagogical module for the process of weaning from mechanical ventilation in advanced nursing education within a cardiothoracic intensive care unit in Malaysia. The authors assess effectiveness using pre test and post test theoretical scores and qualitative feedback from instructors and students. I have evaluated the manuscript according to the official PLOS ONE criteria.

**Reviewer #2:**

Comments

•The manuscript repeatedly claims the use of a regression discontinuity design (RDD). However, the actual implementation does not meet the essential methodological criteria of RDD, including:

No modeling of the running variable

No graphical or regression-based discontinuity analysis

No local linear regression or bandwidth justification

Arbitrary cut-off score (70) without theoretical or empirical justification

What is presented is effectively a non-randomized pre–post quasi-experimental study, not a true RDD.

Recommendation:

Either reframe the design explicitly as a quasi-experimental pre–post study, or properly implement and report RDD analysis following established guidelines.

Failure to correct this will be considered a methodological misrepresentation.

•Total sample size is extremely small (N = 24), with an imbalanced control group (n = 5).

No power analysis or justification for sample adequacy is provided.

Claims of effectiveness and generalizability are not supported by this sample size.

•The manuscript repeatedly reports p = .000, which is statistically incorrect.

Recommendation:

Replace all instances of “p = .000” with p < 0.001

Emphasize effect sizes and confidence intervals, not only p-values

•Control Group Analysis Is Inadequate

Although a control group is mentioned:

No clear between-group comparison is presented

Analyses focus almost exclusively on within-group pre–post changes

Control group outcomes are underreported and analytically underused

•Reliability indices (e.g., Cronbach’s alpha)

Whether the instruments truly measure critical thinking, decision-making, and judgment?

Provide detailed psychometric evidence or clearly state that the measures assess knowledge performance, not higher-order cognitive skills.

•The manuscript contains:

Frequent grammatical errors

Redundant phrasing

Long, repetitive paragraphs

Informal or non-academic expressions

Recommendation:

Substantial English language editing by a professional academic editor is required.

•The Author Summary section reads as a professional biography rather than a scientific summary, which is not aligned with PLOS ONE conventions.

Recommendation:

Remove biographical content and Focus on study rationale, methods, and key findings

•Ethics approval is mentioned but inconsistently reported across sections

Data availability statement contradicts PLOS ONE policy by implying restricted access

Recommendation:

Harmonize ethics statements across manuscript and submission form . Clearly state how and where anonymized data can be accessed, or justify restrictions.

**Reviewer #3:**

Thank you for the opportunity to review this manuscript. The study addresses an important educational gap in advanced nursing practice and provides preliminary evidence that a structured pedagogical module may improve nurses’ knowledge and decision‑making regarding weaning from mechanical ventilation.

What are the main claims of the paper and how significant are they for the discipline?

The present study develops and implements a pedagogical module for the process of mechanical ventilation weaning (WPMV) in a cardiothoracic ICU (ICU). It uses a quasi-experimental design (discontinuous regression), in which there is a significant improvement in knowledge/skills, critical thinking and decision-making in the intervention group (n=19), with an increase in average grade from 52.9% to 90.5% (p≤0.001). The topic is relevant for nursing education in critical care.

QUESTIONS:

1. Are the claims properly placed in the context of the previous literature? Have the authors treated the literature fairly?

The manuscript presents a theoretical pedagogical framework (Johns, Taba, Gagné) and cites studies and clinical guides related to the subject of study. However, it is proposed that the authors add in the text why the design used for the study is suitable and how it aligns with the methodological literature.

2. Do the data and analyses fully support the claims? If not, what other evidence is required?

The study uses a cutoff point 70 in a pretest made before the intervention, to create "weak" (n=19) and "skillful/control" (n=5) groups. Since the analyses are pre-post with paired samples, it is proposed to the authors the implementation of comparisons between both groups and adjustment by confusing.

On the other hand, the authors used Kolmogorov-Smirnov and Shapiro-Wilk for the determination of normality. Being a small sample (n<50 or n<30), the test of choice would be Shapiro-Wilk for its greater power and precision. In this case, the authors confirm the normality of the sample (p>0.05), but in Table 8: normality test, it is indicated that the Shapiro-Wilk test (pretest) shows p=0.015 (not normal), which contradicts the assumption of normality and requires the use of non-parametric tests.

It is also proposed to the authors if it is possible to detail the instruments used in the training (exam specifications/skills, validity, reliability, rubrics, items, examples)

3. PLOS ONE encourages authors to publish detailed protocols and algorithms as supporting information online. Do any particular methods used in the manuscript warrant such treatment? If a protocol is already provided, for example for a randomized controlled trial, are there any important deviations from it? If so, have the authors explained adequately why the deviations occurred?

The documents provided as supplementary material justify the methodology used in the development of the study.

4. If the paper is considered unsuitable for publication in its present form, does the study itself show sufficient potential that the authors should be encouraged to resubmit a revised version?

Yes. The manuscript shows a clear design, solid statistical results and a relevant educational contribution to advanced training in CICU nursing. The document demonstrates clear potential for publication if reviewed.

5. Are original data deposited in appropriate repositories and accession/version numbers provided for genes, proteins, mutants, diseases, etc.?

The authors indicate that the data is stored privately and securely.

6. Does the study conform to any relevant guidelines such as CONSORT, MIAME, QUORUM, STROBE, and the Fort Lauderdale agreement?

No, the study does not declare adherence to any of these guidelines.

7. Are details of the methodology sufficient to allow the experiments to be reproduced?

Partially. It would be missing to implement the aspects that are requested in the review to provide the study with greater methodological clarity.

8. Is any software created by the authors freely available?

It does not apply.

9. Is the manuscript well organized and written clearly enough to be accessible to non-specialists?

Yes.

10. Is it your opinion that this manuscript contains an NIH-defined experiment of Dual Use concern?

No.

**********

6. PLOS authors have the option to publish the peer review history of their article (what does this mean?). If published, this will include your full peer review and any attached files.

If you choose “no”, your identity will remain anonymous but your review may still be made public.

**Do you want your identity to be public for this peer review?**  For information about this choice, including consent withdrawal, please see our Privacy Policy.

Reviewer #1: No

Reviewer #2: No

Reviewer #3: No

**********

[NOTE: If reviewer comments were submitted as an attachment file, they will be attached to this email and accessible via the submission site. Please log into your account, locate the manuscript record, and check for the action link "View Attachments". If this link does not appear, there are no attachment files.]

To ensure your figures meet our technical requirements, please review our figure guidelines: https://journals.plos.org/plosone/s/figures

You may also use PLOS’s free figure tool, NAAS, to help you prepare publication quality figures: https://journals.plos.org/plosone/s/figures#loc-tools-for-figure-preparation.

NAAS will assess whether your figures meet our technical requirements by comparing each figure against our figure specifications.

Attachment Submitted filename: renamed_f80ce.docx

# Author comment: Author response to Decision Letter 1

**DOI:** 10.1371/journal.pone.0332792.r002

4 Apr 2026

Dear PLOS ONE Editorial Staff,

Thank you for the technical assessment of our manuscript. We have addressed all formatting and structural issues raised by the journal office to comply with PLOS ONE submission guidelines.

Response to Editorial Technical Issues:

1. Supporting Information Captions

Issue: Missing captions for Supporting Information files at the end of the manuscript.

Revision: We have added a new section titled "Supporting Information" at the very end of the manuscript (after the References). It includes a caption for the raw data: "S1 Data. Anonymized raw data for theoretical pre-test and post-test scores." We have also added an in-text citation in the Methodology section to refer to this file.

2. File Format

Issue: Manuscript not in an accepted file type.

Revision: We have ensured the manuscript is submitted as a standard Microsoft Word (.docx) file. We have removed any previous PDF versions of the manuscript from the submission system.

3. Reference to Figures 3 and 4

Issue: Figures 3 and 4 were not cited in the text.

Revision: We have added explicit citations for Fig 3 in the "Module Development" section and Fig 4 in the "Data Collection/Implementation" section to ensure the reader is correctly directed to these visuals.

4. Separate Captions for Each Figure

Issue: Missing separate captions for each figure within the manuscript.

Revision: We have inserted clear, separate text captions for Fig 1, Fig 2, Fig 3, and Fig 4 directly within the manuscript document. Please note that the actual image files have been uploaded separately as high-resolution TIFF/EPS files, as per guidelines.

5. Reference to Table 4

Issue: Table 4 was not mentioned in the text.

Revision: We have added a sentence in the Results section (Case Processing Summary) that explicitly refers to Table 4 to ensure proper production linking.

Sincerely,

Dr. Sakinah Awangharun

Corresponding Author

Medical Education Research and Development Unit

Faculty of Medicine, University of Malaya

Attachment Submitted filename: Response to Reviewers_PONE-D-25-48105.docx

# Peer review: Decision Letter 1

**DOI:** 10.1371/journal.pone.0332792.r003

**Authors:** Javier Fagundo-Rivera

24 Apr 2026

PONE-D-25-48105R1

Effectiveness of a Pedagogical Module for The Process of Weaning from Mechanical Ventilation in Advanced Nursing Education

PLOS One

Dear Dr. AWANGHARUN,

Thank you for submitting your manuscript to PLOS ONE. After careful consideration, we feel that it has merit but does not fully meet PLOS ONE’s publication criteria as it currently stands. Therefore, we invite you to submit a revised version of the manuscript that addresses the points raised during the review process.

==============================

**ACADEMIC EDITOR:**

Dear Authors,

Thank you for submitting the revised version of your manuscript following the first round of external peer review.

After careful evaluation, it is evident that the revision remains incomplete. Specifically, you have not provided responses to any of the comments raised by Reviewer 1, and several comments from Reviewer 2 also remain unaddressed. Furthermore, Reviewer 3 has expressed dissatisfaction with the responses concerning the statistical aspects of the study. The issues identified raise substantive concerns regarding the integrity and robustness of the methodology, as the detected errors may significantly affect the validity of the study’s findings.

We are therefore returning the manuscript to you for a further and final round of revision. Please be advised that, should the revised submission fail to adequately address all reviewer concerns, rejection of the manuscript will be strongly considered.

We urge you to carefully and thoroughly respond to each reviewer comment explicitly and individually. Please avoid summarizing or merging responses, as clear, point-by-point replies greatly facilitate the review process and ensure transparency in your revisions.

We look forward to receiving your revised manuscript.

Sincerely,

Academic Editor

==============================

Please submit your revised manuscript by Jun 08 2026 11:59PM. If you will need more time than this to complete your revisions, please reply to this message or contact the journal office at plosone@plos.org. When you're ready to submit your revision, log on to https://www.editorialmanager.com/pone/ and select the 'Submissions Needing Revision' folder to locate your manuscript file.

Please include the following items when submitting your revised manuscript:

- A letter that responds to each point raised by the academic editor and reviewer(s). You should upload this letter as a separate file labeled 'Response to Reviewers'.
- A marked-up copy of your manuscript that highlights changes made to the original version. You should upload this as a separate file labeled 'Revised Manuscript with Track Changes'.
- An unmarked version of your revised paper without tracked changes. You should upload this as a separate file labeled 'Manuscript'.

If you would like to make changes to your financial disclosure, please include your updated statement in your cover letter. Guidelines for resubmitting your figure files are available below the reviewer comments at the end of this letter.

If applicable, we recommend that you deposit your laboratory protocols in protocols.io to enhance the reproducibility of your results. Protocols.io assigns your protocol its own identifier (DOI) so that it can be cited independently in the future. For instructions see: https://journals.plos.org/plosone/s/submission-guidelines#loc-laboratory-protocols. Additionally, PLOS ONE offers an option for publishing peer-reviewed Lab Protocol articles, which describe protocols hosted on protocols.io. Read more information on sharing protocols at https://plos.org/protocols?utm_medium=editorial-email&utm_source=authorletters&utm_campaign=protocols.

As the corresponding author, your ORCID iD is verified in the submission system and will appear in the published article. PLOS supports the use of ORCID, and we encourage all coauthors to register for an ORCID iD and use it as well. Please encourage your coauthors to verify their ORCID iD within the submission system before final acceptance, as unverified ORCID iDs will not appear in the published article. *Only*  the individual author can complete the verification step; PLOS staff *cannot*  verify ORCID iDs on behalf of authors.

We look forward to receiving your revised manuscript.

Kind regards,

Javier Fagundo-Rivera, PhD

Academic Editor

PLOS One

Journal Requirements:

If the reviewer comments include a recommendation to cite specific previously published works, please review and evaluate these publications to determine whether they are relevant and should be cited. There is no requirement to cite these works unless the editor has indicated otherwise.

**Additional Editor Comments:**

Dear Authors,

Thank you for submitting the revised version of your manuscript following the first round of external peer review.

After careful evaluation, it is evident that the revision remains incomplete. Specifically, you have not provided responses to any of the comments raised by Reviewer 1, and several comments from Reviewer 2 also remain unaddressed. Furthermore, Reviewer 3 has expressed dissatisfaction with the responses concerning the statistical aspects of the study. The issues identified raise substantive concerns regarding the integrity and robustness of the methodology, as the detected errors may significantly affect the validity of the study’s findings.

We are therefore returning the manuscript to you for a further and final round of revision. Please be advised that, should the revised submission fail to adequately address all reviewer concerns, rejection of the manuscript will be strongly considered.

We urge you to carefully and thoroughly respond to each reviewer comment explicitly and individually. Please avoid summarizing or merging responses, as clear, point-by-point replies greatly facilitate the review process and ensure transparency in your revisions.

We look forward to receiving your revised manuscript.

Sincerely,

Academic Editor

[Note: HTML markup is below. Please do not edit.]

Reviewers' comments:

Reviewer's Responses to Questions

**Comments to the Author**

1. If the authors have adequately addressed your comments raised in a previous round of review and you feel that this manuscript is now acceptable for publication, you may indicate that here to bypass the “Comments to the Author” section, enter your conflict of interest statement in the “Confidential to Editor” section, and submit your "Accept" recommendation.

Reviewer #1: All comments have been addressed

Reviewer #3: All comments have been addressed

**********

2. Is the manuscript technically sound, and do the data support the conclusions?

The manuscript must describe a technically sound piece of scientific research with data that supports the conclusions. Experiments must have been conducted rigorously, with appropriate controls, replication, and sample sizes. The conclusions must be drawn appropriately based on the data presented.

Reviewer #1: Partly

Reviewer #3: (No Response)

**********

3. Has the statistical analysis been performed appropriately and rigorously?

Reviewer #1: Yes

Reviewer #3: (No Response)

**********

4. Have the authors made all data underlying the findings in their manuscript fully available?

The PLOS Data policy requires authors to make all data underlying the findings described in their manuscript fully available without restriction, with rare exception (please refer to the Data Availability Statement in the manuscript PDF file). The data should be provided as part of the manuscript or its supporting information, or deposited to a public repository. For example, in addition to summary statistics, the data points behind means, medians and variance measures should be available. If there are restrictions on publicly sharing data—e.g. participant privacy or use of data from a third party—those must be specified.

Reviewer #1: No

Reviewer #3: (No Response)

**********

5. Is the manuscript presented in an intelligible fashion and written in standard English?

PLOS ONE does not copyedit accepted manuscripts, so the language in submitted articles must be clear, correct, and unambiguous. Any typographical or grammatical errors should be corrected at revision, so please note any specific errors here.

Reviewer #1: No

Reviewer #3: (No Response)

**********

6. Review Comments to the Author

Please use the space provided to explain your answers to the questions above. You may also include additional comments for the author, including concerns about dual publication, research ethics, or publication ethics. (Please upload your review as an attachment if it exceeds 20,000 characters)

**Reviewer #1:**

This manuscript presents the development and evaluation of a pedagogical module for the weaning process from mechanical ventilation in advanced nursing education within a cardiothoracic intensive care unit context. The study addresses an important topic in clinical education and provides original data derived from a structured educational intervention. The revised version shows improvement in several technical and reporting aspects. However, significant methodological and interpretative concerns remain before the manuscript can be considered for publication.

**Major Comments**

***Alignment of Conclusions with Evidence***

The conclusions extend beyond the data presented. The manuscript claims that the pedagogical module contributes to reducing clinical complications and legal risks. These outcomes were not measured in the study. The data are limited to theoretical test scores and qualitative feedback. The conclusions should be restricted to educational outcomes such as knowledge acquisition and perceived improvement in cognitive skills. Statements related to clinical outcomes and legal implications should be removed or clearly reframed as speculative.

***Study Design and Group Allocation***

Participants were assigned to groups using a cut off score of 70 determined by the researcher. This threshold remains insufficiently justified. There is no theoretical or empirical rationale provided to support this decision. This raises concerns about internal validity and potential bias in group allocation. A clearer justification is required, or the limitation should be explicitly acknowledged and discussed.

In addition, the control group consists of only five participants. This severely limits the strength of any comparative interpretation. The manuscript should address the implications of this imbalance and avoid overinterpretation of differences between groups.

***Statistical Analysis and Interpretation***

The inclusion of additional analyses such as the Wilcoxon Signed Rank Test and effect size calculations represents an improvement. However, these results are not adequately interpreted. Effect sizes are reported but not discussed in terms of magnitude or practical significance. Confidence intervals are presented in tables but are not integrated into the narrative.

The analysis remains focused on within group comparisons. The absence of robust between group analysis limits the strength of the conclusions. The manuscript would benefit from a clearer explanation of the analytical strategy and its limitations.

***Conceptual Clarity and Measurement***

Key constructs such as critical thinking, decision making, and clinical judgment are central to the study but remain insufficiently defined. The manuscript does not clearly explain how these constructs were operationalized or measured within the theoretical tests and assessments. Clear definitions and alignment between constructs and measurement tools are necessary to support the validity of the findings.

***Discussion and Interpretation***

The Discussion section remains largely descriptive and reiterates results rather than providing critical interpretation. There is limited engagement with existing literature on nursing education and mechanical ventilation training. The authors should strengthen this section by contextualizing their findings within current evidence and by discussing alternative explanations.

Limitations are not sufficiently developed. The manuscript should explicitly address the small sample size, single center design, non randomized allocation, short duration of follow up, and reliance on theoretical outcomes.

***Data Availability and Compliance***

The data availability statement remains unclear and does not fully comply with journal policy. The manuscript states that data are stored securely by the authors, which suggests restricted access. At the same time, it indicates that data are available within the manuscript and supporting files. This inconsistency should be resolved. The authors must provide a clear statement ensuring that data are accessible without restriction or justify any limitations in accordance with journal guidelines.

***Language and Presentation***

The manuscript remains intelligible but requires further language editing. Grammatical errors and awkward phrasing are still present. Professional editing is recommended to improve clarity and readability. Repetition across sections should be reduced, particularly in the Results and Discussion.

**Minor Comments**

Some terminology is used inconsistently and should be standardized throughout the manuscript. Tables and figures would benefit from clearer labeling and more concise captions. The Methods section could be streamlined to improve readability.

**Reviewer #2: comments that have not been responded.**

•Control Group Analysis Is Inadequate

Although a control group is mentioned:

No clear between-group comparison is presented

Analyses focus almost exclusively on within-group pre–post changes

Control group outcomes are underreported and analytically underused

•Reliability indices (e.g., Cronbach’s alpha)

Whether the instruments truly measure critical thinking, decision-making, and judgment?

Provide detailed psychometric evidence or clearly state that the measures assess knowledge performance, not higher-order cognitive skills.

**Reviewer #3:**  (No Response) **ACADEMIC EDITOR COMMENTS: Please, revise this comment by reviewer 3.**

1) The study uses a cutoff point 70 in a pretest made before the intervention, to create "weak" (n=19) and "skillful/control" (n=5) groups. Since the analyses are pre-post with paired samples, it is proposed to the authors the implementation of comparisons between both groups and adjustment by confusing.

2) On the other hand, the authors used Kolmogorov-Smirnov and Shapiro-Wilk for the determination of normality. Being a small sample (n<50 or n<30), the test of choice would be Shapiro-Wilk for its greater power and precision. In this case, the authors confirm the normality of the sample (p>0.05), but in Table 8: normality test, it is indicated that the Shapiro-Wilk test (pretest) shows p=0.015 (not normal), which contradicts the assumption of normality and requires the use of non-parametric tests.

3) It is also proposed to the authors if it is possible to detail the instruments used in the training (exam specifications/skills, validity, reliability, rubrics, items, examples).

**********

7. PLOS authors have the option to publish the peer review history of their article (what does this mean?). If published, this will include your full peer review and any attached files.

If you choose “no”, your identity will remain anonymous but your review may still be made public.

**Do you want your identity to be public for this peer review?**  For information about this choice, including consent withdrawal, please see our Privacy Policy.

Reviewer #1: No

Reviewer #3: No

**********

[NOTE: If reviewer comments were submitted as an attachment file, they will be attached to this email and accessible via the submission site. Please log into your account, locate the manuscript record, and check for the action link "View Attachments". If this link does not appear, there are no attachment files.]

To ensure your figures meet our technical requirements, please review our figure guidelines: https://journals.plos.org/plosone/s/figures

You may also use PLOS’s free figure tool, NAAS, to help you prepare publication quality figures: https://journals.plos.org/plosone/s/figures#loc-tools-for-figure-preparation.

NAAS will assess whether your figures meet our technical requirements by comparing each figure against our figure specifications.

Attachment Submitted filename: renamed_c4ca4.docx

# Author comment: Author response to Decision Letter 2

**DOI:** 10.1371/journal.pone.0332792.r004

12 May 2026

Date: May 6, 2026

To: The Editor, PLOS ONE

Subject: Resubmission of Manuscript [PONE-D-25-48105]

Title: Effectiveness of a Pedagogical Module for The Process of Weaning from Mechanical Ventilation in Advanced Nursing Education"

Dear Editor and Reviewers,

We would like to express our sincere gratitude for the constructive feedback provided on our manuscript. We have performed a major revision to address each of the technical and structural concerns raised. Below is our point-by-point response to the reviewers' comments:

This manuscript presents the development and evaluation of a pedagogical module for the weaning process from mechanical ventilation in advanced nursing education within a cardiothoracic intensive care unit context. The study addresses an important topic in clinical education and provides original data derived from a structured educational intervention. The revised version shows improvement in several technical and reporting aspects. However, significant methodological and interpretative concerns remain before the manuscript can be considered for publication.

Reviewer #1 Comments

No Major Comments Amendment Page/Level

1 Alignment of Conclusions with Evidence

The conclusions extend beyond the data presented. The manuscript claims that the pedagogical module contributes to reducing clinical complications and legal risks. These outcomes were not measured in the study. The data are limited to theoretical test scores and qualitative feedback. The conclusions should be restricted to educational outcomes such as knowledge acquisition and perceived improvement in cognitive skills. Statements related to clinical outcomes and legal implications should be removed or clearly reframed as speculative. We appreciate this important observation. We have revised the conclusion and Author summary to ensure alignment with the study finding. Statement implying direct clinical or legal outcomes have been removed

The conclusions are now strictly limited to educational outcomes, specifically improvements in theoretical knowledge and applied clinical reasoning. References to clinical implications are now clearly framed as potential future impacts rather than demonstrated outcomes. 1 / 23 -45

27/ 724-745

2 Study Design and Group Allocation

Participants were assigned to groups using a cut off score of 70 determined by the researcher. This threshold remains insufficiently justified. There is no theoretical or empirical rationale provided to support this decision. This raises concerns about internal validity and potential bias in group allocation. A clearer justification is required, or the limitation should be explicitly acknowledged and discussed.

In addition, the control group consists of only five participants. This severely limits the strength of any comparative interpretation. The manuscript should address the implications of this imbalance and avoid overinterpretation of differences between groups.

Thank you for this valuable feedback. We have clarified that the 70% cut-off score reflects the institutional competency benchmark used in the study setting for advanced clinical assessments.

We have also revised the manuscript to clearly describe the study as a quasi-experimental pre–post design without randomisation, where the comparison group serves as a performance benchmark rather than a basis for inferential comparison.

Furthermore, we have explicitly acknowledged the small and unequal group sizes as a limitation and reframed the study as a pilot pedagogical evaluation within a specialised clinical context.

7-9/ 191-248

3 Statistical Analysis and Interpretation

The inclusion of additional analyses such as the Wilcoxon Signed Rank Test and effect size calculations represents an improvement. However, these results are not adequately interpreted. Effect sizes are reported but not discussed in terms of magnitude or practical significance. Confidence intervals are presented in tables but are not integrated into the narrative.

The analysis remains focused on within group comparisons. The absence of robust between group analysis limits the strength of the conclusions. The manuscript would benefit from a clearer explanation of the analytical strategy and its limitations. We have revised the Results section to include interpretation of effect sizes using Cohen’s criteria. The findings now explicitly indicate a large educational effect, demonstrating practical significance beyond statistical significance.

Additionally, 95% confidence intervals have been integrated into the narrative to improve clarity regarding the precision of estimates.

We have also clarified that the primary analysis focuses on within-group changes, and the limitations of between-group comparisons are now explicitly discussed. 12-18/ 298-408

4 Conceptual Clarity and Measurement

Key constructs such as critical thinking, decision making, and clinical judgment are central to the study but remain insufficiently defined. The manuscript does not clearly explain how these constructs were operationalized or measured within the theoretical tests and assessments. Clear definitions and alignment between constructs and measurement tools are necessary to support the validity of the findings.

We have strengthened the conceptual framework by providing clear definitions of critical thinking, decision-making, and clinical judgment, supported by relevant literature.

In the Methods section, we have clarified that these constructs were operationalised using case-based clinical scenarios, requiring participants to analyse patient data, make decisions, and justify clinical actions. To improve conceptual accuracy, we now describe these outcomes as applied clinical reasoning.

7-9/ 191-248

5 Discussion and Interpretation

The Discussion section remains largely descriptive and reiterates results rather than providing critical interpretation. There is limited engagement with existing literature on nursing education and mechanical ventilation training. The authors should strengthen this section by contextualizing their findings within current evidence and by discussing alternative explanations.

Limitations are not sufficiently developed. The manuscript should explicitly address the small sample size, single center design, non randomized allocation, short duration of follow up, and reliance on theoretical outcomes.

We have substantially revised the Discussion section to provide a more critical and analytical interpretation of findings.

The revised discussion:

Contextualises results within existing literature on simulation-based and structured nursing education

Addresses the theory–practice gap

Introduces a dedicated Strengths and Limitations subsection

Discusses alternative explanations and implications for practice 21-27/ 469 - 735

6 Data Availability and Compliance

The data availability statement remains unclear and does not fully comply with journal policy. The manuscript states that data are stored securely by the authors, which suggests restricted access. At the same time, it indicates that data are available within the manuscript and supporting files. This inconsistency should be resolved. The authors must provide a clear statement ensuring that data are accessible without restriction or justify any limitations in accordance with journal guidelines.

We have revised the Data Availability Statement to ensure full compliance with the journal’s policy and to resolve any perceived inconsistency. We confirm that all data required to replicate the study’s findings more specifically the anonymized pre- and post-test scores and qualitative feedback are now included as Supporting Information. The previous reference to restricted storage by the authors has been removed to guarantee unrestricted access to the minimal data set while maintaining strict participant confidentiality 12-21

300-468

7 Language and Presentation

The manuscript remains intelligible but requires further language editing. Grammatical errors and awkward phrasing are still present. Professional editing is recommended to improve clarity and readability. Repetition across sections should be reduced, particularly in the Results and Discussion.

We have conducted a comprehensive linguistic and structural review to enhance clarity and professional tone. Specifically, we have focused on refining grammatical. Reducing redundancy and clarity 1-30 /

1-757

8 Minor Comments

Some terminology is used inconsistently and should be standardized throughout the manuscript. Tables and figures would benefit from clearer labelling and more concise captions. The Methods section could be streamlined to improve readability. We have performed a thorough review to standardize all terminology, all table labels and figure and the method section 1-30 / 1-757

Reviewer #2: comments that have not been responded.

No Major Comments Amendment Page/Level

1 •Control Group Analysis Is Inadequate

Although a control group is mentioned:

No clear between-group comparison is presented

Analyses focus almost exclusively on within-group pre–post changes

Control group outcomes are underreported and analytically underused We have clarified that due to the small sample size (n = 5) and non-randomised allocation, robust inferential comparison was not feasible.

We have included descriptive comparisons to show that the control group maintained high baseline performance, while the intervention group demonstrated substantial improvement. The control group is now explicitly described as a performance benchmark. 12-18/ 298-408

2 •Reliability indices (e.g., Cronbach’s alpha)

Whether the instruments truly measure critical thinking, decision-making, and judgment?

Provide detailed psychometric evidence or clearly state that the measures assess knowledge performance, not higher-order cognitive skills. We acknowledge the importance of this issue. We have revised the manuscript to clarify that the assessment measures knowledge performance and applied clinical reasoning through structured case-based scenarios.

Given the nature of the instrument and study design, we have avoided overstating measurement of higher-order constructs and have refined the terminology accordingly. 12-18/ 298-408

Reviewer #3: (No Response) ACADEMIC EDITOR COMMENTS: Please, revise this comment by reviewer 3. - All revised and amended

No Major Comments Amendment Page/Level

1 The study uses a cutoff point 70 in a pretest made before the intervention, to create "weak" (n=19) and "skilful/control" (n=5) groups. Since the analyses are pre-post with paired samples, it is proposed to the authors the implementation of comparisons between both groups and adjustment by confusing. We appreciate this suggestion. While we have included descriptive comparisons between groups, inferential adjustment for confounders was not feasible due to the small and unequal sample sizes.

We have explicitly acknowledged this limitation and clarified that the study’s primary objective is to evaluate within-group improvement following the intervention. 12-18/ 298-408

2 On the other hand, the authors used Kolmogorov-Smirnov and Shapiro-Wilk for the determination of normality. Being a small sample (n<50 or n<30), the test of choice would be Shapiro-Wilk for its greater power and precision. In this case, the authors confirm the normality of the sample (p>0.05), but in Table 8: normality test, it is indicated that the Shapiro-Wilk test (pretest) shows p=0.015 (not normal), which contradicts the assumption of normality and requires the use of non-parametric tests. We thank the reviewer for identifying the contradiction in our normality reporting. Upon re-examination of Table 8, we acknowledge that the Shapiro-Wilk p-value (p=0.015) indicates a non-normal distribution for the pre-test data. Following the reviewer's recommendation, we have standardized our reporting to prioritize the Shapiro-Wilk test given our sample size (n < 50). Consequently, we have corrected the manuscript to reflect that non-parametric tests is specifically the Wilcoxon Signed-Rank Test were used for all pre-post comparisons. This change ensures our analytical strategy is statistically sound and properly aligned with the distribution of our data, thereby increasing the reliability of our conclusions 12-18/ 298-408

3 It is also proposed to the authors if it is possible to detail the instruments used in the training (exam specifications/skills, validity, reliability, rubrics, items, examples). We have expanded the 'Materials and Methods' section and added a new Supporting Information file (S2 Appendix) to include the Exam Specifications. This includes a breakdown of how test items map to specific cognitive domains (Knowledge, Critical Thinking, and Decision-Making), examples of case-based items used, and the rubric employed for qualitative feedback. Furthermore, we have detailed the content validation process by the expert panel and provided the reliability indices (Shapiro-Wilk and Cronbach’s alpha) as discussed in previous points. This additional detail ensures the transparency of our measurement tools and supports the validity of our educational findings 12-18/ 298-408

Sincerely,

Dr. Sakinah Awang @ Harun

Corresponding Author

University of Malaya, Malaysia

Attachment Submitted filename: Response to Reviewers 3_PONE-D-25-48105.docx

# Peer review: Decision Letter 2

**DOI:** 10.1371/journal.pone.0332792.r005

**Authors:** Javier Fagundo-Rivera

28 May 2026

<div>PONE-D-25-48105R2

Effectiveness of a Pedagogical Module for The Process of Weaning from Mechanical Ventilation in Advanced Nursing Education

PLOS One

Dear Dr. AWANGHARUN,

Thank you for submitting your manuscript to PLOS ONE. After careful consideration, we feel that it has merit but does not fully meet PLOS ONE’s publication criteria as it currently stands. Therefore, we invite you to submit a revised version of the manuscript that addresses the points raised during the review process.

==============================

**ACADEMIC EDITOR:**

Dear Authors,

Thank you for submitting the revised version of your manuscript, “Effectiveness of a Pedagogical Module for The Process of Weaning from Mechanical Ventilation in Advanced Nursing Education” (Manuscript Number: PONE-D-25-48105R2).

The reviewers acknowledge the substantial improvements made in response to the previous comments. In particular, they appreciate the clarification of the quasi-experimental design, the inclusion of additional statistical analyses, the improved discussion of limitations, and the more cautious interpretation of the educational outcomes. The manuscript addresses an important topic in advanced nursing education and is now considerably closer to publishable form.

However, several points still require attention before the manuscript can be considered for acceptance:

* Please reconsider the use of the term “control group,” as reviewers consider this concept methodologically questionable in the context of the current design. Terms such as “comparison group” may be more appropriate.

* Some sections, particularly the Author Summary, Introduction, and Discussion, still contain statements suggesting improvements in patient safety, reduction of complications, or direct clinical impact. These claims should be revised to remain fully aligned with the educational scope of the study and the acknowledged limitations.

* Please further moderate any overinterpretation of cognitive or clinical implications.

* The English language and overall writing quality should be improved, especially in the Discussion and Author Summary sections.

* Please consider explicitly including the main study limitations in the abstract.

In light of these remaining issues, the decision is Minor Revision.

We look forward to receiving your revised manuscript.

Best regards.

==============================

Please submit your revised manuscript by Jul 12 2026 11:59PM. If you will need more time than this to complete your revisions, please reply to this message or contact the journal office at plosone@plos.org. When you're ready to submit your revision, log on to https://www.editorialmanager.com/pone/ and select the 'Submissions Needing Revision' folder to locate your manuscript file.

Please include the following items when submitting your revised manuscript:

- A letter that responds to each point raised by the academic editor and reviewer(s). You should upload this letter as a separate file labeled 'Response to Reviewers'.
- A marked-up copy of your manuscript that highlights changes made to the original version. You should upload this as a separate file labeled 'Revised Manuscript with Track Changes'.
- An unmarked version of your revised paper without tracked changes. You should upload this as a separate file labeled 'Manuscript'.

If you would like to make changes to your financial disclosure, please include your updated statement in your cover letter. Guidelines for resubmitting your figure files are available below the reviewer comments at the end of this letter.

If applicable, we recommend that you deposit your laboratory protocols in protocols.io to enhance the reproducibility of your results. Protocols.io assigns your protocol its own identifier (DOI) so that it can be cited independently in the future. For instructions see: https://journals.plos.org/plosone/s/submission-guidelines#loc-laboratory-protocols. Additionally, PLOS ONE offers an option for publishing peer-reviewed Lab Protocol articles, which describe protocols hosted on protocols.io. Read more information on sharing protocols at https://plos.org/protocols?utm_medium=editorial-email&utm_source=authorletters&utm_campaign=protocols.

As the corresponding author, your ORCID iD is verified in the submission system and will appear in the published article. PLOS supports the use of ORCID, and we encourage all coauthors to register for an ORCID iD and use it as well. Please encourage your coauthors to verify their ORCID iD within the submission system before final acceptance, as unverified ORCID iDs will not appear in the published article. *Only*  the individual author can complete the verification step; PLOS staff *cannot*  verify ORCID iDs on behalf of authors.

We look forward to receiving your revised manuscript.

Kind regards,

Javier Fagundo-Rivera, PhD

Academic Editor

PLOS One

Journal Requirements:

If the reviewer comments include a recommendation to cite specific previously published works, please review and evaluate these publications to determine whether they are relevant and should be cited. There is no requirement to cite these works unless the editor has indicated otherwise.

Please review your reference list to ensure that it is complete and correct. If you have cited papers that have been retracted, please include the rationale for doing so in the manuscript text, or remove these references and replace them with relevant current references. Any changes to the reference list should be mentioned in the rebuttal letter that accompanies your revised manuscript. If you need to cite a retracted article, indicate the article’s retracted status in the References list and also include a citation and full reference for the retraction notice.

**Additional Editor Comments:**

Dear Authors,

Thank you for submitting the revised version of your manuscript, “Effectiveness of a Pedagogical Module for The Process of Weaning from Mechanical Ventilation in Advanced Nursing Education” (Manuscript Number: PONE-D-25-48105R2).

The reviewers acknowledge the substantial improvements made in response to the previous comments. In particular, they appreciate the clarification of the quasi-experimental design, the inclusion of additional statistical analyses, the improved discussion of limitations, and the more cautious interpretation of the educational outcomes. The manuscript addresses an important topic in advanced nursing education and is now considerably closer to publishable form.

However, several points still require attention before the manuscript can be considered for acceptance:

* Please reconsider the use of the term “control group,” as reviewers consider this concept methodologically questionable in the context of the current design. Terms such as “comparison group” may be more appropriate.

* Some sections, particularly the Author Summary, Introduction, and Discussion, still contain statements suggesting improvements in patient safety, reduction of complications, or direct clinical impact. These claims should be revised to remain fully aligned with the educational scope of the study and the acknowledged limitations.

* Please further moderate any overinterpretation of cognitive or clinical implications.

* The English language and overall writing quality should be improved, especially in the Discussion and Author Summary sections.

* Please consider explicitly including the main study limitations in the abstract.

In light of these remaining issues, the decision is Minor Revision.

We look forward to receiving your revised manuscript.

Best regards.

[Note: HTML markup is below. Please do not edit.]

Reviewers' comments:

Reviewer's Responses to Questions

**Comments to the Author**

1. If the authors have adequately addressed your comments raised in a previous round of review and you feel that this manuscript is now acceptable for publication, you may indicate that here to bypass the “Comments to the Author” section, enter your conflict of interest statement in the “Confidential to Editor” section, and submit your "Accept" recommendation.

Reviewer #1: All comments have been addressed

Reviewer #3: All comments have been addressed

**********

2. Is the manuscript technically sound, and do the data support the conclusions?

The manuscript must describe a technically sound piece of scientific research with data that supports the conclusions. Experiments must have been conducted rigorously, with appropriate controls, replication, and sample sizes. The conclusions must be drawn appropriately based on the data presented.

Reviewer #1: Partly

Reviewer #3: Partly

**********

3. Has the statistical analysis been performed appropriately and rigorously?

Reviewer #1: Yes

Reviewer #3: No

**********

4. Have the authors made all data underlying the findings in their manuscript fully available?

The PLOS Data policy requires authors to make all data underlying the findings described in their manuscript fully available without restriction, with rare exception (please refer to the Data Availability Statement in the manuscript PDF file). The data should be provided as part of the manuscript or its supporting information, or deposited to a public repository. For example, in addition to summary statistics, the data points behind means, medians and variance measures should be available. If there are restrictions on publicly sharing data—e.g. participant privacy or use of data from a third party—those must be specified.

Reviewer #1: Yes

Reviewer #3: Yes

**********

5. Is the manuscript presented in an intelligible fashion and written in standard English?

PLOS ONE does not copyedit accepted manuscripts, so the language in submitted articles must be clear, correct, and unambiguous. Any typographical or grammatical errors should be corrected at revision, so please note any specific errors here.

Reviewer #1: No

Reviewer #3: Yes

**********

6. Review Comments to the Author

Please use the space provided to explain your answers to the questions above. You may also include additional comments for the author, including concerns about dual publication, research ethics, or publication ethics. (Please upload your review as an attachment if it exceeds 20,000 characters)

**Reviewer #1: SEE DOCUMENT ATTACHED**

The revised manuscript shows clear improvement compared with the previous submission. Several methodological and reporting concerns have been addressed appropriately, particularly regarding clarification of the quasi-experimental design, inclusion of additional statistical analyses, improved acknowledgment of study limitations, and a more cautious interpretation of findings. The study addresses an important topic in advanced nursing education and provides potentially valuable preliminary findings within a specialized clinical training context. However, several concerns remain partially unresolved, particularly regarding internal validity, conceptual overinterpretation of cognitive constructs, residual overstatement of clinical implications, and language quality. The manuscript is closer to publishable form, but additional moderate revision would further strengthen its methodological clarity and interpretive rigor.

**Reviewer #3:**

The authors have adequately responded to most key criticisms: The conclusions are now limited to educational results (knowledge and clinical reasoning), the study is now correctly presented as non-randomised pre-post quasi-experimental, the work is explicitly recognised as a pedagogical pilot study, includes explicit limitations (n small, a centre, non-randomised, etc.).

However, the manuscript presents methodologically relevant weaknesses, such as the design and sample size, where the "control group" remains conceptually questionable. This concept can be reformed by "comparison group" or similar.

On the other hand, drafting inconsistencies remain, since in several sections (Author summary, introduction and discussion) sentences still appear that suggest the improvement of patient safety, the reduction of complications and the clinical impact of the study, which partially contradicts the corrections already made by the authors.

It is also recommended to improve the writing of English, especially in the discussion and Author summary.

Likewise, it may be relevant to clarify the limitations of the study in the abstract.

**********

7. PLOS authors have the option to publish the peer review history of their article (what does this mean?). If published, this will include your full peer review and any attached files.

If you choose “no”, your identity will remain anonymous but your review may still be made public.

**Do you want your identity to be public for this peer review?**  For information about this choice, including consent withdrawal, please see our Privacy Policy.

Reviewer #1: No

Reviewer #3: No

**********

[NOTE: If reviewer comments were submitted as an attachment file, they will be attached to this email and accessible via the submission site. Please log into your account, locate the manuscript record, and check for the action link "View Attachments". If this link does not appear, there are no attachment files.]

To ensure your figures meet our technical requirements, please review our figure guidelines: https://journals.plos.org/plosone/s/figures

You may also use PLOS’s free figure tool, NAAS, to help you prepare publication quality figures: https://journals.plos.org/plosone/s/figures#loc-tools-for-figure-preparation.

NAAS will assess whether your figures meet our technical requirements by comparing each figure against our figure specifications.

Attachment Submitted filename: renamed_587b2.docx

# Author comment: Author response to Decision Letter 3

**DOI:** 10.1371/journal.pone.0332792.r006

1 Jun 2026

Date: May 29, 2026

To: The Editor, PLOS ONE

Subject: Resubmission of Manuscript [PONE-D-25-48105]

Title: Effectiveness f a Pedagogical Module for The Process of Weaning from Mechanical Ventilation in Advanced Nursing Education"

Dear Editor and Reviewers,

We would like to express our sincere gratitude for the constructive feedback provided on our manuscript. We have performed a major revision to address each of the technical and structural concerns raised. Below is our point-by-point response to the reviewers' comments:

This manuscript presents the development and evaluation of a pedagogical module for the weaning process from mechanical ventilation in advanced nursing education within a cardiothoracic intensive care unit context. The study addresses an important topic in clinical education and provides original data derived from a structured educational intervention. The revised version shows improvement in several technical and reporting aspects. However, significant methodological and interpretative concerns remain before the manuscript can be considered for publication.

Reviewer #1 Comments

Minor Comments Amendment Page/Level

1. Alignment of Conclusions with Evidence

The revision has improved the alignment between the conclusions and the evidence presented. The abstract and conclusion now focus primarily on educational outcomes such as theoretical knowledge and applied clinical reasoning.

However, some statements within the manuscript still imply direct clinical impact that was not measured in the study. For example, references to improving patient safety, optimizing the weaning process, and reducing medico-legal risks remain present in sections such as the Author Summary and Introduction. These claims go beyond the available evidence and should either be removed or clearly framed as speculative future implications. We thank the reviewer for this constructive guidance. We completely agree that this study directly evaluated educational and cognitive outcomes via a pre- and post-test design, rather than tracing longitudinal, patient-level clinical metrics (such as actual mechanical ventilation days or direct malpractice claim rates).

We wish to clarify that patient safety and risk mitigation were actively evaluated within our instrument by operationalizing these concepts through higher-order cognitive scenarios. In alignment with established frameworks like Tanner's Model of Clinical Judgment and Bloom's Taxonomy, our assessment items required students to perform clinical application, data analysis, and synthesis of safety parameters (e.g., calculating the Rapid Shallow Breathing Index and determining Spontaneous Breathing Trial readiness). Therefore, an increase in these scores directly demonstrates a significant improvement in the nurses' capacity to maintain patient safety.

However, to ensure absolute clarity and avoid any perception of overstating our baseline data, we have thoroughly revised the text in the Author Summary, 1) Introduction, 2) Future Recommendations, and 3) Conclusion

All references to patient safety, weaning optimization, and medico-legal risk reduction have been re-framed explicitly as prospective, long-term educational goals and potential clinical implications rather than immediate clinical outcomes. 2/ 61-63, 80 -84, 4/ 133-135, 2-25/ 11 – 687

2. Study Design and Group Allocation

The explanation regarding the use of the 70% competency benchmark has improved, and the authors now clarify that the study should be interpreted as a quasi-experimental pilot educational evaluation rather than a randomized controlled comparison.

The manuscript also acknowledges the limitation of the small and unequal comparison group. This represents an important improvement.

Nevertheless, the methodological concern regarding group allocation remains only partially resolved. The intervention group consisted mainly of lower-performing participants, whereas the comparison group consisted of higher-performing participants at baseline. This creates a considerable risk of regression-to-the-mean effects and limits the interpretability of comparative outcomes.

In addition, the statement indicating that the cut-off score “was determined by the researcher herself” remains problematic and should be revised or more rigorously justified. We thank the reviewer for this excellent methodological critique. We recognize that our original phrasing (“was determined by the researcher herself”) incorrectly implied personal subjectivity. We have completely revised this across the manuscript to reflect the strict, objective institutional framework of our design.

1. Rigorous Justification of the 70% Cut-Off: The phrase “determined by the researcher herself” has been removed from the manuscript. We have corrected the Participants and Sampling section to clarify that 70% is the official, pre-existing absolute competency cutoff mandated by the Nursing Division and Medical Education unit at the National Heart Institute (IJN) for specialized credentialing. In advanced clinical education, using a fixed percentage threshold is a standard, ubiquitous practice. It directly mirrors international lifesaver and advanced nursing certification frameworks (such as the American Heart Association's cognitive cut-scores for BLS/ACLS courses or advanced nurse credentialing exams), where a fixed score ranging from 70% to 84% is utilized as an absolute baseline to ensure minimum cognitive safety.

2. Mitigation and Defense Against Regression-to-the-Mean (RTM) Confounders: The reviewer raises an important point regarding selection bias when isolating lower-performing candidates. To address this concern with full transparent scientific rigor, we have contextualized our work within Benjamin Bloom's Criterion-Referenced Mastery Learning (CRML) educational model. In specialized units like the Cardiothoracic Intensive Care Unit (CICU), it is an ethical and structural necessity to focus intensive educational interventions precisely on the group of staff performing below safe operational thresholds.

While statistical regression can cause mild positive changes, it is mathematically incapable of generating the profound changes captured by our evaluation instruments:

• The intervention group achieved a massive 37.63-point mean score leap ( p <0.001).

• The paired sample test yielded a substantial t statistic of 14.30.

• Most importantly, the calculated Cohen's d effect size is 3.28.

According to established educational evaluation literature (e.g., Hanley & Hutson, 2019), a Cohen's exceeding completely outstrips the maximum variance attributable to statistical regression artifacts, demonstrating true educational gain. We have added a dedicated sub-section to our Limitations explicitly detailing the allocation structure, small/unequal group parameters, and RTM dynamics to prevent over-interpretation of comparative outcomes, fully aligning with the reviewer's instructions.

9/ 220-232

11/ 289-301

3 Statistical Analysis and Interpretation

The statistical reporting has improved substantially. The addition of Wilcoxon Signed-Rank testing, confidence intervals, and effect size estimates strengthens the analytical rigor of the study. The interpretation of effect sizes is also clearer than in the previous version.

The manuscript appropriately clarifies that between-group comparisons are descriptive rather than inferential because of the small and imbalanced sample sizes.

Some inconsistencies in the analytical presentation remain. Parametric paired t-tests continue to be emphasized despite evidence of non-normality in the pre-test scores. The statistical narrative could still be streamlined to improve coherence and reduce redundancy. Despite this, the overall concern has been addressed satisfactorily. We sincerely thank the reviewer for their valuable guidance regarding the remaining analytical inconsistencies and duplications. We agree completely that presenting non-normal data requires strict alignment between data distribution properties and inferential emphasis. We have extensively overhauled the statistical reporting across the entire manuscript to ensure a streamlined, methodologically rigorous presentation.12-19 312-413

4. Conceptual Clarity and Measurement

The revised manuscript provides improved descriptions of applied clinical reasoning and explains that case-based scenarios were used during assessment. This clarification strengthens the conceptual framework.

However, the operationalization of constructs such as critical thinking, decision-making, and clinical judgment remains insufficiently supported. Although the terminology has been moderated compared with the previous version, the manuscript still presents these constructs as measured outcomes without presenting robust psychometric evidence demonstrating construct validity.

The manuscript would benefit from more consistent terminology emphasizing knowledge performance and applied clinical reasoning rather than validated measurement of higher-order cognitive constructs. We completely agree with the reviewer's excellent psychometric insight. Because our assessment tools were case-based scenario examinations rather than standardized psychometric inventories, claiming to measure independent cognitive constructs was an overstatement.

To align perfectly with the evidence, we have revised the terminology globally throughout the manuscript. We have shifted away from terms like "critical thinking skills" or "clinical judgment metrics" and replaced them with "theoretical knowledge performance" and "applied clinical reasoning scores" (see updates across Abstract, Materials and Methods, Results, and Discussion sections).

Furthermore, we have added an explicit clarification in the Data Analysis and Discussion section noting that our evaluations are domain-specific knowledge application thresholds rather than psychometrically validated cognitive construct measurements.

2-25/ 11 – 687

5. Discussion and Interpretation

The Discussion section has improved considerably and now includes better engagement with relevant educational literature. The addition of a dedicated strengths and limitations subsection strengthens the manuscript and improves transparency.

The limitations related to sample size, non-randomized allocation, single-center design, and reliance on theoretical outcomes are now acknowledged more clearly. We sincerely thank the reviewer for this encouraging feedback. We deeply appreciate your guidance in helping us elevate the scholarly transparency of our manuscript. 19-23

414-578

6. Data Availability and Compliance

The concerns regarding data availability appear to have been adequately resolved. The revised statement is clearer and more consistent with journal policy requirements. We are pleased to hear that the revised Data Availability statement and the inclusion of the raw data repository reference. fully meet the journal’s compliance and policy requirements. All relevant data required to replicate the study findings remain accessible within the manuscript and its supporting information files. Thank you -

7. Language and Presentation

The manuscript is more readable than the previous version. However, substantial grammatical and stylistic issues remain throughout the text. Several sentences still contain awkward phrasing, repetition, and syntactic inconsistencies that reduce readability.

Professional English-language editing remains strongly recommended before publication. We sincerely appreciate the reviewer's attention to the presentation and readability of our manuscript. We have conducted a thorough, line-by-line editorial clean-up of the entire text to address structural phrasing, syntax consistency, and narrative flow.

We thank the reviewer for their careful oversight. We have conducted a final comprehensive text sweep to clean up lingering phrasing redundancies, eliminate run-on sentences in the Abstract, and repair minor parenthetical alignment issues resulting from the previous layout overhaul. Most importantly, a text description discrepancy under the normality section was completely rectified to align explicitly with our non-parametric Wilcoxon analysis and the actual data trends shown in our tables. The entire document is now fully harmonized to PLOS ONE's high standards of clarity and rigor. 2-25/ 11 – 687

Reviewer 3;

Minor Comments Amendment Page/Level

1. The authors have adequately responded to most key criticisms: The conclusions are now limited to educational results (knowledge and clinical reasoning), the study is now correctly presented as non-randomised pre-post quasi-experimental, the work is explicitly recognised as a pedagogical pilot study, includes explicit limitations (n small, a centre, non-randomised, etc.).

However, the manuscript presents methodologically relevant weaknesses, such as the design and sample size, where the "control group" remains conceptually questionable. This concept can be reformed by "comparison group" or similar.

On the other hand, drafting inconsistencies remain, since in several sections (Author summary, introduction and discussion) sentences still appear that suggest the improvement of patient safety, the reduction of complications and the clinical impact of the study, which partially contradicts the corrections already made by the authors.

It is also recommended to improve the writing of English, especially in the discussion and

Author summary.

Likewise, it may be relevant to clarify the limitations of the study in the abstract. Amended. We completely agree with this stylistic refinement. We have gone through the entire manuscript and replaced the phrase "control group" with "comparison group" across all relevant narratives, ensuring the nomenclature accurately reflects our quasi-experimental pilot design.

Amended. We have carefully scrubbed the text to eliminate any direct assertions of direct clinical impact or active error prevention.

In the Introduction, the sentence regarding legal issues was rewritten to clarify that this is a "proactive educational strategy designed to minimize underlying factors associated with future risks" .

In the Discussion, similar corrections were applied to maintain a strict focus on classroom educational performance metrics.

Amended. A thorough proofreading and line-by-line editorial polish was completed across the text. We specifically targeted and completely removed several large blocks of text that had been accidentally duplicated back-to-back during a previous copy-paste compilation phase (specifically deleting duplicate statistical descriptions in the Methods section and repeated challenge blocks in the Discussion section) . Broken parenthetical phrasing has been fixed to ensure smooth academic readability.

Amended. We have updated the Abstract (Methods/Results/Conclusion)

We appreciate the reviewer's meticulous validation of our non-randomized quasi-experimental pilot parameters. A final targeted text search was executed, and the term "Control Group" has been completely purged from the document, including lingering table title strings within the Supporting Information listings at the very end of the text layout. The comparison cohort is now correctly and consistently identified across all sections of the submission. 2-25/ 11 – 687, 19-23, 414-578

23-25, 579-687

Attachment Submitted filename: Response_to_Reviewers_3_PONE-D-25-48105_auresp_3.docx

# Editor report: Decision Letter 3

**DOI:** 10.1371/journal.pone.0332792.r007

**Authors:** Javier Fagundo-Rivera

2 Jun 2026

Effectiveness of a Pedagogical Module for The Process of Weaning from Mechanical Ventilation in Advanced Nursing Education

PONE-D-25-48105R3

Dear Dr. AWANGHARUN,

We’re pleased to inform you that your manuscript has been judged scientifically suitable for publication and will be formally accepted for publication once it meets all outstanding technical requirements.

Within one week, you’ll receive an e-mail detailing the required amendments. When these have been addressed, you’ll receive a formal acceptance letter and your manuscript will be scheduled for publication.

An invoice will be generated when your article is formally accepted. Please note, if your institution has a publishing partnership with PLOS and your article meets the relevant criteria, all or part of your publication costs will be covered. Please make sure your user information is up-to-date by logging into Editorial Manager at Editorial Manager® and clicking the ‘Update My Information' link at the top of the page. For questions related to billing, please contact billing support.

If your institution or institutions have a press office, please notify them about your upcoming paper to help maximize its impact. If they’ll be preparing press materials, please inform our press team as soon as possible -- no later than 48 hours after receiving the formal acceptance. Your manuscript will remain under strict press embargo until 2 pm Eastern Time on the date of publication. For more information, please contact onepress@plos.org.

Kind regards,

Javier Fagundo-Rivera, PhD

Academic Editor

PLOS One

**Additional Editor Comments:**

Dear Authors,

We are pleased to inform you that your manuscript PONE-D-25-48105R3: "Effectiveness of a Pedagogical Module for The Process of Weaning from Mechanical Ventilation in Advanced Nursing Education" has been accepted for publication in PLOS ONE.

The editors and reviewers have carefully evaluated your revised submission and are satisfied that the manuscript meets the journal's standards for publication. We appreciate your thoughtful responses to the reviewers' comments and the revisions made throughout the review process.

Your manuscript will now proceed to the production stage. You will receive further communications regarding copyediting, proof review, and publication scheduling from the PLOS ONE production team.

Thank you for choosing PLOS ONE for the publication of your work. We look forward to seeing your article published and contributing to the scientific literature.

Congratulations on the acceptance of your manuscript.

Sincerely,

Javier Fagundo-Rivera, PhD

Academic Editor

PLOS ONE

Reviewers' comments: -

# Editor report: Acceptance letter

**DOI:** 10.1371/journal.pone.0332792.r008

**Authors:** Javier Fagundo-Rivera

PONE-D-25-48105R3

PLOS One

Dear Dr. AWANGHARUN,

I'm pleased to inform you that your manuscript has been deemed suitable for publication in PLOS One. Congratulations! Your manuscript is now being handed over to our production team.

At this stage, our production department will prepare your paper for publication. This includes ensuring the following:

* All references, tables, and figures are properly cited

* All relevant supporting information is included in the manuscript submission,

* There are no issues that prevent the paper from being properly typeset

You will receive further instructions from the production team, including instructions on how to review your proof when it is ready. Please keep in mind that we are working through a large volume of accepted articles, so please give us a few days to review your paper and let you know the next and final steps.

Lastly, if your institution or institutions have a press office, please let them know about your upcoming paper now to help maximize its impact. If they'll be preparing press materials, please inform our press team within the next 48 hours. Your manuscript will remain under strict press embargo until 2 pm Eastern Time on the date of publication. For more information, please contact onepress@plos.org.

You will receive an invoice from PLOS for your publication fee after your manuscript has reached the completed accept phase. If you receive an email requesting payment before acceptance or for any other service, this may be a phishing scheme. Learn how to identify phishing emails and protect your accounts at https://explore.plos.org/phishing.

If we can help with anything else, please email us at customercare@plos.org.

Thank you for submitting your work to PLOS ONE and supporting open access.

Kind regards,

PLOS ONE Editorial Office Staff

on behalf of

Dr. Javier Fagundo-Rivera

Academic Editor

PLOS One
